# Prospects of HIV elimination among men who have sex with men: A systematic review of modeling studies

**DOI:** 10.1371/journal.pcbi.1014596

**Published:** 2026-08-31

**Authors:** Jacob Aiden Roberts, Alexandra Teslya, Mirjam E. Kretzschmar, Janneke H.H.M. van de Wijgert, Ganna Rozhnova

**Affiliations:** 1 Julius Center for Health Sciences and Primary Care, University Medical Center Utrecht, Utrecht University, Utrecht, The Netherlands; 2 Center for Complex Systems Studies (CCSS), Utrecht University, Utrecht, The Netherlands; 3 Institute of Epidemiology and Social Medicine, University of Münster, Münster, Germany; 4 Faculty of Sciences, BioISI—Biosystems & Integrative Sciences Institute, University of Lisbon, Lisbon, Portugal; 5 Faculty of Sciences, University of Lisbon, Lisbon, Portugal; The Second Xiangya Hospital of Central South University, CHINA

## Abstract

Men who have sex with men (MSM) remain disproportionately affected by HIV worldwide. This systematic review summarizes the results of mathematical modeling studies that evaluated prospects of HIV elimination among MSM by geographical setting, type of intervention(s), elimination definition, and model characteristics. We searched Embase and PubMed for studies published between July 1, 2016 and September 1, 2025 which used a dynamic mathematical model to assess the impact of interventions on HIV transmission among MSM. Data were extracted on study population, interventions, elimination definitions, model type, model structure, and calibration. Studies were critically appraised for model comprehensiveness in addressing elimination. 135 of the 4,595 records were included. MSM populations in six of the eight Joint United Nations Programme on HIV/AIDS regions were modeled, with 47% of models considering MSM in the USA. Agent-based models (ABMs) were as common as compartmental models overall, with ABMs more frequently used in Western and Central Europe and North America (WCENA), while compartmental models predominated elsewhere. Of the 135 included studies, 41 defined elimination, and they defined it as follows: (i) reduction in HIV incidence/prevalence, or (ii) threshold of HIV incidence/prevalence, or (iii) reproduction number below one. Elimination was achieved in 42 out of 51 modeled scenarios, of which 32 (82.05%) were in WCENA, but the authors of only 28 of these 42 scenarios discussed the real-world elimination feasibility with 10 of these scenarios considered elimination feasible by the original authors. There was also a strong regional divide in the elimination scenarios considered feasible, with 6 (60.00%) in Asia and the Pacific (AP) and 4 (40.00%) in WCENA. Models in which elimination was achieved commonly used combinations of interventions. Modeling efforts to understand HIV elimination prospects outside WCENA should be intensified, and models assessing HIV elimination prospects should account for HIV acquisition outside of the local context. To enhance study comparability and ensure that models contribute effectively to public health policy, an elimination definition based on an HIV incidence threshold would be the most valuable. By identifying gaps in current studies, we recommend novel research directions for modeling to inform a coordinated global response for HIV elimination among MSM.

## Author summary

Men who have sex with men (MSM) continue to experience a disproportionate burden of HIV worldwide. The Joint United Nations Programme on HIV/AIDS (UNAIDS) strategy to end the AIDS epidemic by 2030 has intensified treatment and prevention efforts, yet it remains unclear whether these are sufficient to achieve HIV elimination in MSM. Public health decisions often rely on mathematical models, which estimate how different interventions might affect HIV transmission. We systematically reviewed dynamic modeling studies published between July 2016 and September 2025 to assess whether proposed interventions could eliminate HIV in MSM across different countries, intervention types, elimination definitions, and modeling approaches. Across studies, combinations of interventions were more likely than single interventions to achieve elimination under conditions that could realistically be implemented. Pre-exposure prophylaxis featured prominently in these scenarios, often alongside increased HIV testing and/or higher antiretroviral therapy coverage. Our review identified important research gaps, including limited modeling in many UNAIDS regions outside of Western and Central Europe and North America. Additionally, we found that studies used different definitions of elimination, making it difficult to compare results. To support clearer interpretation and more coordinated decision-making, we recommend using a common definition of elimination based on an HIV incidence threshold.

## Introduction

The HIV epidemic remains a major public health problem, particularly among key populations [1]. The Joint United Nations Programme on HIV/AIDS (UNAIDS) considers men who have sex with men (MSM) as one of the main key populations vulnerable to HIV acquisition and transmission. Despite considerable progress in HIV prevention and treatment overall, MSM continue to have a disproportionately high HIV incidence and prevalence worldwide [1]. In 2022, global HIV prevalence among MSM was eleven times higher than among adults in the general population [2]. Alarmingly, the annual number of new HIV infections among MSM increased by 11% globally and by 19% outside sub-Saharan Africa from 2010 to 2022 [3]. Contrasting trends in reaching elimination among MSM are currently observed in different UNAIDS regions. Some countries in Western and Central Europe and North America are characterized by a rapidly declining epidemic (e.g., [4–6]), while emerging and ongoing HIV epidemics are reported in the Middle East and North Africa [7,8], the Caribbean [9], and no evidence of slowing epidemics is found in Africa [10].

To address a disproportionate burden of HIV among MSM, interventions such as classical partner reduction and condom use approaches, pre-exposure prophylaxis (PrEP) [11], and test-and-treat [12] are used either separately or in combination to reach this key population [13]. Predicting the impact of interventions on HIV dynamics at the population level empirically is challenging. Mathematical modeling can guide the design of interventions and plays an increasingly important role in supporting evidence-based policymaking in public health [14]. Models describe transmission dynamics using equations and/or computer simulations. Modeling studies are often the only way to investigate large-scale complex HIV dynamics, particularly in cases where experiments are not ethical or logistically impossible. A well-designed model can assist policymakers in making decisions on HIV control and elimination.

Despite a large body of modeling studies that investigate the impact of interventions on HIV transmission dynamics [15], including the assessment of HIV elimination strategies [4,16], the literature concerning the prospects of HIV elimination among MSM worldwide is inconsistent. The success of HIV elimination in a specific context depends on a combination of factors, such as the target population, the state of the HIV epidemic, HIV care and prevention practices, and details of sexual behavior that shape HIV transmission among MSM. Among regions where the HIV epidemic is generalized to the heterosexual population, such as sub-Saharan Africa, interventions are often targeted at key populations other than MSM. These intervention packages are often successful in reducing overall HIV incidence, however the proportion of infections among MSM in these regions continues to increase [3], demonstrating the importance of considering MSM as a key-population in the context of HIV elimination globally. Evaluation of HIV elimination prospects is complicated by the fact that different authors use different definitions of elimination, posing a barrier to a unified response. HIV elimination is usually considered accomplished upon reaching a certain quantifiable threshold, often based on guidelines issued by (inter)national public health authorities. Definitions of elimination include achieving zero new HIV infections, reducing HIV incidence to a low level, or reaching a point where the HIV epidemic is no longer a public health threat [17]. A 90% reduction in HIV incidence by 2030 is an example of the latter definition used in ending the HIV epidemic goals in the USA [18].

The elimination definition of fewer than one HIV infection per 1,000 persons per year was adopted from the seminal modeling study by Granich et al. [12], which received attention from the public health community by demonstrating the possibility of HIV elimination.

This systematic review aims to improve our understanding of the prospects of HIV elimination among MSM globally. We choose to focus on MSM as this subpopulation experiences a greatly disproportionate burden of HIV compared to its population size, with MSM experiencing a 26 times greater risk of acquiring HIV compared to the general population [19]. Eliminating HIV in all populations necessitates eliminating HIV in key subpopulations such as MSM, even in regions where the HIV epidemic is more generalized. HIV incidence in subpopulations such as MSM has been underestimated in these generalized regions, and focus has shifted away from them, leaving them left-behind [20]. We summarized mathematical modeling studies by (i) geographical setting where elimination may or may not be achieved, (ii) elimination definitions used, and (iii) interventions required to achieve elimination. We discuss the knowledge gaps in these areas and identify further modeling research needed to better inform policy about effective intervention strategies for ultimately achieving the ambitious goal of HIV elimination among MSM, contributing significantly towards HIV elimination programs overall.

## Methods

This systematic review adhered to PRISMA guidelines [21]. The full PRISMA checklist is available in S1 Table. The data extraction file is available in S1 Data. A list of inclusion and exclusion reasons for all searched articles is provided in S2 Table. No protocol was registered for this review. Article exclusion criteria were defined prior to screening.

### Search strategy and selection criteria

Embase and PubMed were searched for studies published between July 1, 2016 and September 1, 2025, when the search was conducted. The starting date was chosen to coincide with the publication of the World Health Organization (WHO) consolidated guidelines on the use of antiretroviral drugs for treating and preventing HIV infection [22]. The search string was (“HIV” OR “human immunodeficiency virus”) AND (“homosexual*” OR “transgender*” OR “gay” OR “MSM” OR “men who have sex with men” OR “men having sex with men” OR “bisexual*” OR “key popu*” OR “key group*” OR “risk group*” OR “vulnerable popu*” OR “vulnerable group*” OR “affected popu*” OR “affected group*” OR “high-risk popu*” OR “high-risk group*” OR “at-risk popu*” OR “at-risk group*” OR “marginialized popu*” OR “marginalized group*”) AND (“model*” OR “framework” OR “simulat*”) AND (“treat*” OR “prevent*”) AND (“mathematic*” OR “transm*” OR “comput*”).

Studies were included if they (i) involved a dynamic model for HIV transmission, where the force of infection depends on the state of the population at a given time, and (ii) assessed the impact of interventions on HIV transmission among MSM. Studies for HIV transmission in a broader population involving MSM were included if they reported a direct or indirect impact of interventions on HIV outcomes among MSM specifically. Studies that involved a dynamic co-transmission model of HIV and another sexually transmitted infection (STI) were included if the primary outcome was HIV. As we were interested in the epidemiological impact of interventions assessed using dynamic transmission models, statistical, back-calculation and decision-analytic models were excluded. Studies focused on methodology rather than the impact of interventions on HIV transmission were excluded. In order to focus our review on HIV outcomes among MSM, studies were included only if they reported HIV outcomes in MSM disaggregated, rather than only as a part of the general population. This particularly applies to studies modeling mixed populations such as cost-effectiveness studies or those focusing on HIV contexts where the epidemiology is more dispersed among populations other than MSM, such as in sub-Saharan Africa. Conference abstracts, reviews, preprints, and articles without full text and articles published outside the specified time range were also excluded.

### Data extraction and analysis

Two authors independently screened the titles and abstracts for inclusion and identified eligible studies using Rayyan software. Three authors independently conducted full-text screening and extracted data using a predefined data extraction form. Study inclusion was by consensus. Rare disagreements were resolved through detailed discussions of the studies in question. The authors regularly compared extracted data to ensure consistency in the review process. Data were extracted on article information, study population, interventions, elimination definition, model type, model structure, and calibration (43 data fields in total; S1 Data). Data fields were summarized descriptively unless quantitative data were available.

Studies were categorized geographically by country and by UNAIDS region (Asia and the Pacific, Caribbean, Eastern and Southern Africa, Eastern Europe and Central Asia, Latin America, Middle East and North Africa, Western and Central Africa, Western and Central Europe and North America) [2]. Additionally, studies were categorized by characteristics of the modeled population, such as demographics (age and ethnicity), subgroups (MSM only or MSM and other subgroups such as the heterosexual population, female sex workers and their clients, injecting drug users, and transgender women), and geographical scale (national, regional, or urban). National scale models consider populations in the entire country. Regional scale models consider a large administrative division within a country (e.g., a state in the USA or a province in China). Urban scale models consider a city or a group of cities and their immediate metropolitan areas. Populations were regarded as stratified by these characteristics if model analyses used different model parameters to describe distinct subgroups.

Models were categorized into deterministic compartmental (DCM), stochastic compartmental (SCM), and agent-based models (ABM). Compartmental models stratify the population into compartments based on certain characteristics, such as disease stage, and track the population in each compartment over time. In contrast, agent-based models include individual heterogeneities and track the status of each individual over time. Unlike deterministic models, stochastic models account for random events. Agent-based models are inherently stochastic.

We reported primary interventions, defined as interventions for which model parameters describing different aspects of intervention engagement (e.g., uptake, retention, coverage, and adherence) and/or intervention efficacy were varied. For example, in a study investigating the increase in PrEP uptake combined with regular HIV testing, PrEP is considered the primary intervention because its uptake was varied. The definition of HIV elimination was a measurable target used in the model analyses to determine when interventions could stop HIV transmission.

Elimination definitions and interventions required to achieve elimination were extracted and narratively reported. We reported the elimination definitions that were assessed in each model. If a model predicted that elimination is achievable, we reported the minimum interventions required; if not, we reported the maximum intervention intensity assessed. For all models, we additionally reported the feasibility of implementing successful elimination scenarios as described by the original study authors. Additionally, for any study in which elimination was deemed technically achievable but not feasible by the original authors, we present the authors’ descriptions of the bottlenecks that would need to be resolved to achieve feasibility.

Additionally, for any study in which elimination was deemed technically achievable but not feasible by the original authors, we present the authors’ descriptions of the bottlenecks that would need to be resolved to achieve feasibility.

Descriptive statistics, i.e., frequencies and percentages, were used to summarize the study locations, characteristics of modeled populations, elimination definitions used, and interventions evaluated. Summary statistics were presented in tables and bar charts. The locations of the studied populations and HIV prevalence were visualized using world maps. Subgroup analyses of the summary statistics were performed to examine differences in elimination prospects, stratified by UNAIDS region, model type, elimination definition, and interventions. All results were pooled quantitatively whenever feasible.

### Critical appraisal

Studies that included an elimination definition were critically appraised by evaluating the comprehensiveness of the models in addressing elimination. Given that different studies pursued different goals, ranging from conceptual analytical investigations to operational modeling, the critical appraisal was not used to exclude studies but rather to evaluate the appropriateness of model structures and methodologies for assessing elimination. In the absence of standardized tools for the critical appraisal of modeling studies focused on elimination, we developed our own scoring system.

The model comprehensiveness score was calculated based on five criteria, such as whether a model accounted for adherence to interventions, sexual risk compensation, the openness of the modeled MSM population (indicating that new HIV infections could be imported or result from sexual contacts with the external population), whether uncertainty in the model outcomes was investigated and reported, and whether a model was validated. S4 Table in S2 Text outlines the questions we used to assess each criterion and provides clear guidance on scoring. Comprehensiveness criteria were chosen to represent core model considerations that we believe should be taken into account in order for a model to accurately assess an intervention’s ability to successfully achieve elimination, see S4 Table in S2 Text for justifications for the chosen criteria. Studies received a score 0, 0.5 or 1 for the inclusion of adherence to interventions, reporting of uncertainty in model outcomes, and model validation. A score of 0 or 1 was assigned for the inclusion of sexual risk compensation and the openness of the MSM population. The individual scores were then normalized and summed to obtain the final score for each study, ranging from 0 to 5, with a higher score indicating that more criteria were satisfied. Critical appraisal was performed independently by two authors. The scoring of studies was by consensus.

It is important to note that this model comprehensiveness score was developed specifically for this review, in order to facilitate a structured comparison of the described criteria. This comprehensiveness score framework has not undergone any formal validation, and should therefore not be interpreted as a widely applicable or validated assessment tool for the comprehensiveness of model considerations for assessing elimination.

### Role of the funding source

The funders of the study had no role in study design, data collection, data analysis, data interpretation, or writing of the report. The corresponding author had full access to all the data in the study and had final responsibility for the decision to submit for publication.

## Results

### Study selection

**Fig 1:**
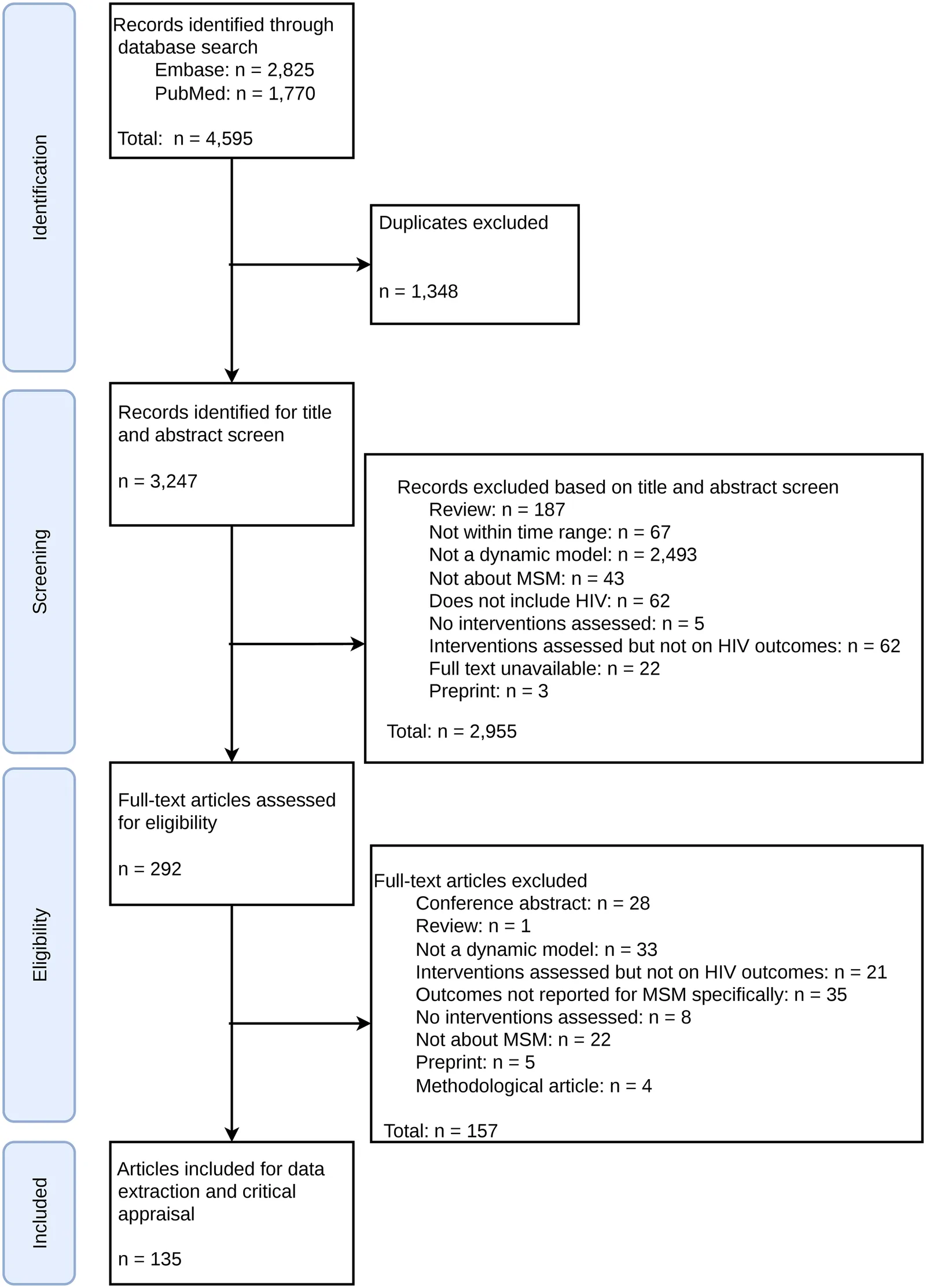
Study selection. MSM = men who have sex with men.

The initial search resulted in 4,595 records, of which 135 studies (2.94%) met the eligibility criteria and were included for data extraction (Fig 1). After removing duplicates and screening titles and abstracts, 4,303 records (93.65%) were excluded. The majority of records excluded based on title and abstract screening were because they did not involve a dynamic transmission model (n = 2,493, 84.37%). Following the full-text assessment, a further 157 of 292 records (53.78%) were excluded; due to not being a dynamic transmission model (n = 33, 21.02%), not reporting outcomes for MSM populations specifically (n = 29, 18.47%, or for other reasons (n = 95, 60.51%). The list of 135 included studies and their characteristics is given in Table 1.

**Table 1:** Characteristics of the included studies. MSM = men who have sex with men. TW = transgender women. PWID = people who inject drugs. FSW = female sex workers. SCM = stochastic compartmental model. DCM = deterministic compartmental model. ABM = agent-based model. IQR = interquartile range.

Authors (Year)	Population	Age (Years)	Location	Model Population Size (Individuals)	Model Type	Cost-effectiveness	HIV Elimination
Adamson et al (2019) [23]	General MSM	15-64	Seattle, USA	45,000	DCM	Yes	No
De Anda et al (2023) [24]	Gay and bisexual MSM	Not specified	Vancouver, Canada	Unclear	SCM	Yes	No
Balasubramanian et al (2022) [25]	African American/Hispanic/Other MSM	≥ 13	32 urban areas of highest HIV burden and Washington DC, USA	Varies by area	SCM	No	No
De Bellis et al (2025) [26]	General MSM	Not specified	The Netherlands	210,000	DCM	No	No
Booton et al (2021) [27]	General MSM	Not specified	Guangzhou, China	12,249-57,856	DCM	No	No
			Shenzhen, China	45,801-180,000			
			Jinan, China	9,687-19,373			
			Qingdao, ChinaQingdao, China	28,000-51,000			
Borquez et al (2020) [28]	MSM + TW + heterosexual population	15-49	Peru	4,767,148 total, 286,028 MSM + TW	DCM	No	No
ten Brink et al (2024) [29]	MSM + FSW + clients + PWID + prisoners + migrants + heterosexual population	All ages	Albania	Unclear	DCM	Yes	No
			Armenia	Unclear			
			Azerbaijan	Unclear			
			Belarus	Unclear			
			Georgia	Unclear			
			Kazakhstan	Unclear			
			Kosovo	Unclear			
			Kyrgyzstan	Unclear			
			Moldova	Unclear			
			Serbia	Unclear			
			Tajikistan	Unclear			
			Uzbekistan	Unclear			
Buchanan et al (2022) [30]	White/African American MSM	18-39	Atlanta-Sandy Springs-Roswell metropolitan area, Georgia, USA	17,440	ABM	No	No
Buchanan et al (2021) [31]	White/African American MSM	18-65	Atlanta, Georgia, USA	11,245	ABM	No	No
Cambiano et al(2023) [32]	Gay, bisexual and other MSM	≥15	UK	Unclear	ABM	Yes	Yes
Cambiano et al (2017) [33]	General MSM	≥15	UK	Unclear	ABM	Yes	No
Chan et al (2019) [34]	General MSM	15-65	Rhode Island, USA	23,815	ABM	No	No
Chen et al (2022) [35]	African American/Hispanic/Other MSM + PWID + heterosexual population	13-64	USA	215,534,336 total, 4,187,426 MSM	DCM	No	Yes
Choi et al (2020) [36]	General MSM	15-64	South Korea	217,280	SCM	Yes	No
David et al (2020) [37]	Gay, bisexual and other MSM	Not specified	Not specified	5,000	DCM	No	Yes
Dimitrov et al (2019a) [38]	MSM + TW + female partners at Harlem site	Not specified	Bangkok, Thailand and Harlem, New York, USA	3,600 men for each site	ABM	No	No
Dimitrov et al (2019b) [39]	MSM + TW	15-49	Lima, Peru	400,000	DCM	No	No
Doyle et al (2023) [40]	MSM	≥15	Montréal, Québec, Canada	10,000	ABM	No	No
Drabo et al (2022) [41]	White/African American/Hispanic MSM	≥15	Los Angeles County, USA	251,521	ABM	No	No
Drabo et al (2016) [42]	General MSM	15-65	Los Angeles Country, USA	176,971	DCM	Yes	No
Elion et al (2019) [43]	African American/Hispanic/White/Other MSM	≥18	USA	4,503,080	ABM	No	No
Estrada et al (2022) [44]	MSM + PWID + heterosexual population	Not specified	Spain	47,141,258 total, 890,200 MSM	DCM	Yes	No
Fojo et al (2021) [45]	African American/Hispanic/Other MSM + heterosexual population + PWID	≥13	32 urban areas of highest HIV burden and Washington DC, USA	Varies by area	DCM	No	Yes
Fraser et al (2021) [46]	MSM + PWID + FSW + clients + heterosexual population	Not specified	Tijuana, Mexico	25,663 total, 5,726 MSM	DCM	No	No
Fraysse et al (2025) [47]	General MSM	≥15	Atlanta, Georiga, USA	10,000	ABM	No	Yes
Gantenberg et al (2018) [48]	General MSM	15-74	Rhode Island, USA	25,000	ABM	No	No
Gilmour et al (2020) [49]	General MSM	18-59	Japan	1,111,426	DCM	No	Yes
Gray (2023) [50]	Gay and bisexual MSM	Not specified	Australia	14,961	DCM	Yes	No
Goedel et al (2020) [51]	General MSM + male sex workers	18-74	Rhode Island, USA	25,000	ABM	Yes	No
Goedel et al (2020) [52]	African American/White MSM	18-39	Atlanta-Sandy Springs-Roswell metropolitan area, Georgia, USA	17,440	ABM	No	No
Goedel et al (2019) [53]	General MSM	15-74	Rhode Island, USA	25,000	ABM	Yes	No
Goedel et al (2018) [54]	African American/White MSM	18-74	Atlanta, Georgia, USA	10,000	ABM	No	No
Goodreau et al (2018) [55]	Adolescent sexual minority MSM	13-18	USA	10,000	ABM	No	No
Gonzalez et al (2025) [56]	MSM + PWID + prisoners + heterosexual populaton	18-80	Spain	156,100 MSM, 3,902,512 total	DCM	Yes	No
Le Guillou et al (2021) [57]	African American/Hispanic/White MSM	15-65	San Francisco, USA	10,000	ABM	No	Yes
Gurski et al (2023) [58]	General MSM	All ages	USA	Unclear (2005 CDC data)	DCM	No	No
Gutowska et al (2022) [59]	General MSM	18-65	USA	7,100,000	DCM	No	Yes
Hamilton et al (2023) [18]	African American/Hispanic/Other MSM + heterosexual population	15-65	16 states and District of Columbia in the South census region, USA	500,000 total, 3.6% MSM	ABM	No	Yes
Hamilton et al (2021a) [60]	General MSM	13-39	Chicago, Illinois, USA	54,000	ABM	No	No
Hamilton et al (2021b) [61]	General MSM	Not specified	Seattle, Washington and Atlanta, Georgia, USA	10,000	ABM	No	No
Hamilton et al (2019) [62]	Adolescent sexual minority MSM + older MSM	13-39	Atlanta, Georgia, USA	13,500	ABM	No	No
Hamilton et al (2018) [63]	African American/White adolescent sexual minority MSM	13-18	Atlanta, Georgia, USA	10,000	ABM	No	No
Hansson et al (2020) [64]	General MSM	16-76	Sweden	Unclear	DCM	No	Yes
Hansson et al (2019) [65]	General MSM	16-76	Stockholm, Sweden	5,000	SCM	No	Yes
He et al (2024) [66]	MSM + FSW + PWID + long distance truck drivers + pregnant women + heterosexual population	15-65	China	Total unclear, 12,000,000 MSM	DCM	Yes	No
Hotton et al (2023) [67]	Young African American MSM	18-34	Arizona, USA	10,000	ABM	No	No
Irvine et al (2020) [68]	Gay and bisexual MSM	Not specified	Greater Vancouver, Canada	20,000	DCM	No	No
Irvine et al (2018) [69]	Gay and bisexual MSM	Not specified	Vancouver, Canada	20,000	SCM	No	No
Jan van Hoek et al (2021) [70]	General MSM	15-64	The Netherlands	20,000	ABM	Yes	No
Jamieson et al (2020) [71]	MSM + FSW + heterosexual population + pregnant women	All ages	South Africa	Unclear	SCM	Yes	No
Jenness et al (2021a) [72]	African American/Hispanic/White/Other MSM	15-65	Atlanta metropolitan area, Georgia, USA	10,000	ABM	No	No
Jenness et al (2021b) [73]	African American/Hispaninc/White/Other MSM	15-65	Atlanta, Georgia, USA	10,000	ABM	Yes	No
Jenness et al (2020) [74]	African American/Hispanic/White MSM	15-65	Atlanta, Georgia, USA	10,000	ABM	No	Yes
Jenness et al (2019) [75]	Young African American/White MSM	18-40	Atlanta, Georgia, USA	10,000	ABM	No	No
Jenness et al (2017) [76]	General MSM	Not specified	USA	Unclear	ABM	No	No
Jenness et al (2016) [77]	General MSM	18-40	USA	10,000	ABM	No	No
Jijon et al (2021) [78]	General MSM	Not specified	Paris region, France	111,000	SCM	No	Yes
Jin et al (2022) [79]	General MSM	≥15	Jiangsu Province, China	723,013	DCM	Yes	No
Jones et al (2022) [80]	African American/Hispanic/White MSM	15-64	Atlanta, Georgia, USA	25,000	ABM	No	No
Kasaie et al (2019) [81]	White/African American MSM	15-75	Baltimore, Maryland, USA	15,000	ABM	No	No
Kasaie et al (2018) [82]	White/African American MSM	15-75	Baltimore, Maryland, USA	15,000	ABM	No	No
Kasaie et al (2017) [83]	White/African American MSM	15-75	Baltimore, Maryland, USA	15,000	ABM	No	No
Kazemian et al (2020) [84]	MSM + TW + PWID	Not specified	India	Unclear	ABM	Yes	No
Khanna et al (2021) [85]	Young African American MSM	18-34	State of Illinois, USA	10,000	ABM	No	Yes
Khanna et al (2019) [86]	Young African American MSM	18-34	State of Illinois, USA	10,000	ABM	No	Yes
Khurana et al (2018) [87]	African American/Hispanic/Other MSM + PWID + heterosexual population	13-64	USA	USA population 2010, 4,187,426 MSM	DCM	No	No
Kusejiko et al (2018) [88]	General MSM	IQR 27–42	Switzerland	5,710	DCM	No	No
Labs et al (2022) [89]	African American MSM	18-64	Jackson, Mississippi, USA	6,825	ABM	No	No
Lee et al (2023) [90]	Young African American MSM	18-34	Houston, Texas, USA	10,000	ABM	No	Yes
Lee et al (2022) [91]	Young African American MSM	18-34	State of Illinois, USA	10,000	ABM	No	Yes
Li et al (2018) [92]	General MSM	15-64	China	3,625,000	DCM	Yes	Yes
Lima et al (2021) [93]	General MSM	15-79	British Columbia, Canada	52,755	DCM	No	Yes
Leng et al (2025) [94]	General MSM	≥18	Cotonou, Benin	1650 - 5445	DCM	Yes	No
LeVasseur et al (2018) [95]	High-risk MSM	Not specified	USA	10,000	ABM	No	No
Lou et al (2018) [96]	General MSM	18-60	Beijing, China	About 11,350	SCM	No	No
Lou et al (2017) [97]	General MSM	15-64	Beijing, China	311,527	DCM	No	Yes
Lyons et al (2022) [98]	General MSM	Not specified	Yaoundé and Douala, Cameroon	Unclear	DCM	No	No
Lu et al (2020) [99]	General MSM	15-64	Zhejiang province, China	25,010	DCM	No	Yes
MacFadden et al (2016) [100]	General MSM	Not specified	Toronto, Canada	57,400	SCM	Yes	No
Maheu-Giroux et al (2017a) [101]	MSM + FSW + clients + general population	15-59	Côte d’Ivoire	2,711,050, 0.8-1.6% MSM	SCM	No	No
Maheu-Giroux et al (2017b) [102]	MSM + FSW + clients + general population	15-59	Côte d’Ivoire	2,711,050, 0.8-1.6% MSM	DCM	No	Yes
Marshall et al (2018) [103]	African American/White MSM	<65	Atlanta, Georgia, USA	11,245	ABM	No	No
Massey et al (2023) [104]	General MSM	Not specified	UK	62,121	SCM	No	Yes
Martin et al (2020) [105]	MSM + general population	Not specified	New York State, USA	Unclear	DCM	No	Yes
McGillen et al (2016) [106]	General MSM + FSW + clients + heterosexual population	≥15	Benin	Unclear	DCM	Yes	No
			Botswana	Unclear			
			Burkina Faso	Unclear			
			Cameroon	Unclear			
			Congo	Unclear			
			DRC	Unclear			
			Ethiopia	Unclear			
			Kenya	Unclear			
			Mali	Unclear			
			Mozambique	Unclear			
			Nigeria	Unclear			
			Rwanda	Unclear			
			Sierra Leone	Unclear			
			South Africa	Unclear			
			Swaziland	Unclear			
			Tanzania	Unclear			
			Zambia	Unclear			
			Zimbabwe	Unclear			
Milwid et al (2022) [107]	Gay and bisexual MSM	≥15	Montreal, Canada	10,000	ABM	No	Yes
Mitchell et al (2023) [108]	African American/White MSM	≥18	Atlanta, Georgia, USA	65,180	DCM	No	Yes
Mitchell et al (2021) [109]	African American/White MSM	≥18	Baltimore, Maryland, USA	65,180	DCM	No	No
Mitchell et al (2019) [110]	African American/White MSM	≥18	Baltimore, Maryland, USA	6,518	DCM	No	Yes
Mitchell et al (2016) [111]	MSM + FSW + clients + general population	Not specified	Bangalore, India	18,367 MSM	DCM	No	Yes
Mukandavire et al (2018) [112]	MSM + FSW + clients + general population	15-49	Dakar, Senegal	895,266 females, 885,570 males with 1.2% MSM	DCM	No	No
Neilan et al (2021) [113]	High-risk young MSM	15	USA	Unclear	ABM	Yes	No
Nichols et al (2016) [114]	General MSM	Not specified	The Netherlands	190,000	DCM	Yes	No
Onwubiko et al (2025) [115]	Gay and bisexual MSM	15-65	Atlanta, Georgia, USA	100,000	ABM	No	No
Palk et al (2018) [4]	General MSM	Not specified	Denmark	54,700	SCM	No	Yes
Reitsema et al (2025) [116]	General MSM	15-64	The Netherlands	20,000	ABM	No	No
Reitsema et al (2024) [117]	General MSM	15-64	The Netherlands	20,000	ABM	No	No
Reitsema et al (2020) [118]	General MSM	15-64	The Netherlands	20,000	ABM	Yes	No
Reitsema et al (2019) [119]	General MSM	15-64	The Netherlands	20,000	ABM	Yes	No
Robineau et al (2017) [120]	High-risk MSM	≥18	Paris, France	10,000	ABM	No	No
Ross et al (2016) [121]	General MSM	Not specified	USA	5,020,000	DCM	Yes	No
Rozhnova et al (2019) [122]	General MSM	Not specified	The Netherlands	Initial size 330,000	DCM	No	No
Rozhnova et al (2018) [16]	General MSM	15-70 years	The Netherlands	Final size 190,000	DCM	No	Yes
Rozhnova et al (2016) [123]	General MSM	15-70 years	The Netherlands	Initial size 330,000	DCM	No	Yes
Silhol et al (2024) [124]	MSM + FSW + clients + general population	15-59	Côte d’Ivoire, Mali, Senegal,	Varies by country	DCM	No	No
Silhol et al (2021a) [125]	MSM + FSW + clients + general population	15-49	Yaoundé, Cameroon	1,217,440, 1.05% MSM	DCM	No	Yes
Silhol et al (2021b) [126]	MSM + FSW + clients + general population	15-49	Yaoundé, Cameroon	1,217,440, 1.05% MSM	DCM	No	No
Silhol et al (2020) [127]	African American/White MSM	≥18	Baltimore, Maryland, USA	6,518	DCM	No	No
Singleton et al (2020) [128]	African American/White MSM	18-39	Atlanta, Georgia, USA	17,440	ABM	No	Yes
Shen et al (2018) [129]	General MSM	18-65	San Francisco, USA	69,122	DCM	Yes	No
Shen et al (2017) [130]	General MSM	18-65	San Francisco, USA	69,122	DCM	No	No
Stansfield et al (2023) [131]	Atlanta: African American/White MSM	Atlanta: ≥18	Atlanta, Georgia, USA	Atlanta: 21500–30000	Atlanta: DCM	No	No
	Montreal: General MSM	Montreal: ≥ 15	Montreal, Canada	Montreal: 10,000	Montreal: ABM		
	The Netherlands: General MSM	The Netherlands: ≥ 15	The Netherlands	The Netherlands: Unclear	The Netherlands: DCM		
Stansfield et al (2021) [132]	General MSM	18-55	USA	10,000	ABM	No	No
Stone et al (2021) [133]	MSM + FSW + clients + general population	15-49	South Africa	MSM are 0.65-7.3% of males	ABM	No	No
Teslya et al (2025) [134]	General MSM	15-75	The Netherlands	25,000	ABM	No	No
Tollet et al (2024) [135]	General MSM	Not specified	Arizona, USA	Non-dimensionalised	DCM	No	Yes
Trickey et al (2022) [136]	General MSM	≥18	Ukraine	181,000	DCM	Yes	No
Ulrich et al (2022) [137]	MSM + TW	15-49	Lima, Peru	127,100	DCM	Yes	No
Vermeer et al (2022) [138]	African American/Hispanic/White MSM	13-70	Chicago, Illinois, USA	6,500	ABM	No	Yes
Versteegh et al (2022) [139]	General MSM	≥15	Thailand	802,470	DCM	Yes	No
Viguerie et al (2024) [140]	Gay, bisexual and other MSM	Not specified	USA	Unclear	DCM	No	No
Van de Vijver et al (2019) [141]	General MSM	≥15	Germany	850,000	DCM	Yes	No
Wang et al (2025) [142]	General MSM	≥15	The Netherlands	163,698	DCM	Yes	Yes
Wang et al (2022) [143]	General MSM	18-59	Japan	1,251,730	DCM	No	Yes
Wang et al (2021) [144]	General MSM	Not specified	Montreal, Toronto, Vancouver, Canada	Varies by city	DCM	No	No
Wang et al (2018) [145]	General MSM	15-64	China	3,600,000	DCM	No	Yes
Wikman-Jorgensen et al (2025) [146]	Gay, bisexual and other MSM	16-85	Spain	892,955	SCM	Yes	No
Wheatly et al (2022) [147]	African American/Hispanic/White MSM	15-64	Atlanta, Georgia, USA	12,000	ABM	Yes	Yes
Wu et al (2025) [148]	General MSM	≥15	Taiwan	218,708	SCM	Yes	Yes
Zang et al (2023) [149]	African American/Hispanic/White MSM + PWID + general population	15-64	Miami-Dade Country, Florida, USA	Unclear	DCM	No	Yes
Zang et al (2022) [150]	General MSM + heterosexual population	15-64	, Atlanta, Georgia, USA	3,586,253, total	SCM	No	No
			Baltimore, Maryland, USA	1,871,812, total			
			Los Angeles, California, USA	6,850,378, total			
			Miami, Florida, USA	1,756,032, total			
			New York City, New York, USA	5,787,014, total			
			Seattle, Washington, USA	1,401,600, total			
Zhang et al (2020) [151]	General MSM	18-65	Beijing, China	Unclear	DCM	No	No
Chen Zhang et al (2019) [152]	General MSM	18-65	Beijing, China	372,634	DCM	Yes	No
Lei Zhang et al (2019) [153]	General MSM	Not specified	China	Unclear	DCM	Yes	No

### Study locations and HIV prevalence

**Fig 2:**
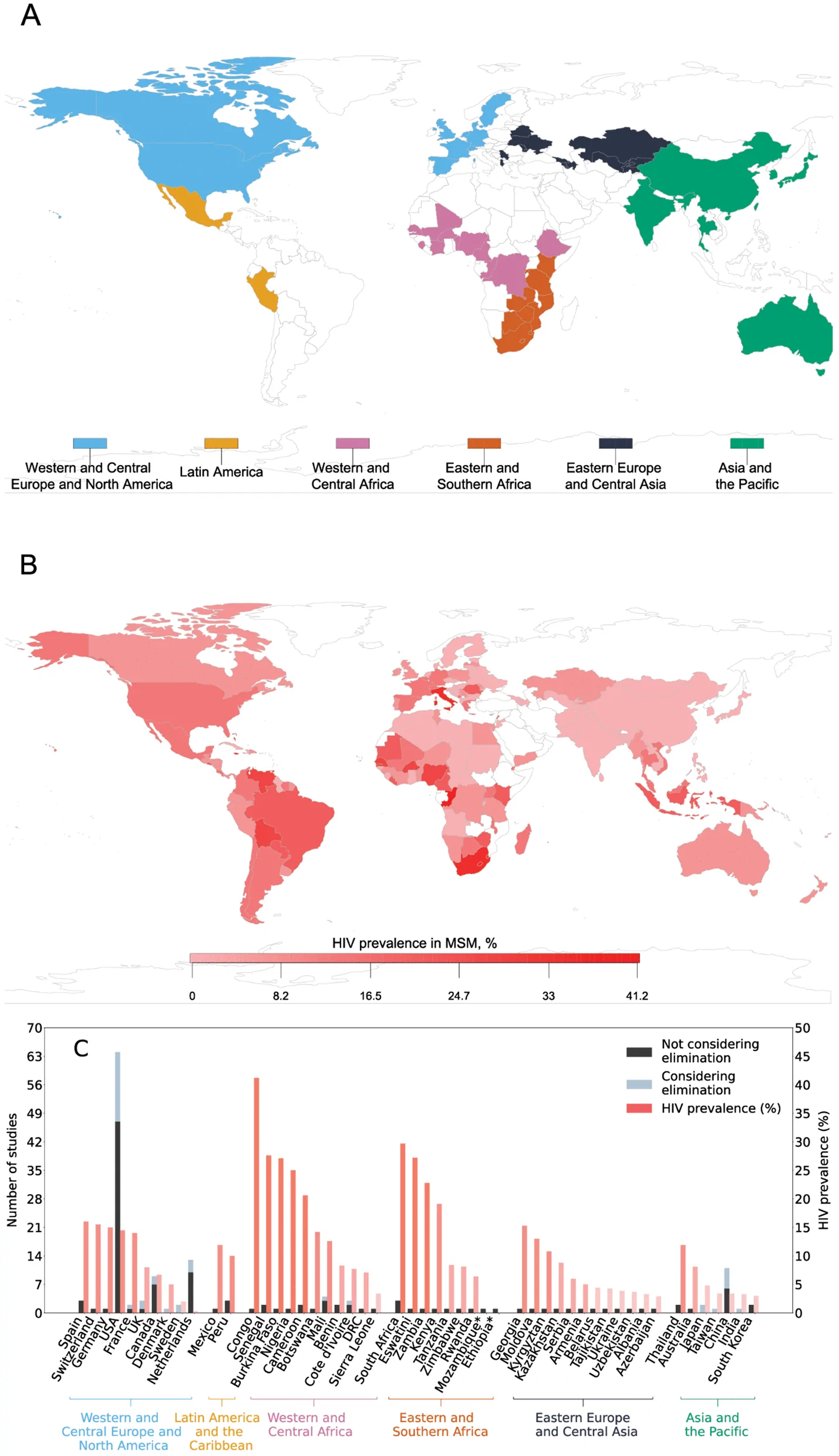
Worldwide location of studies and HIV prevalence among MSM. **(A)** Locations of the modeled populations by UNAIDS region [2]. **(B)** HIV prevalence among MSM by country reported by UNAIDS [1]. In **(A)** and **(B)**, countries with no data are shown in white. **(C)** The number of studies that did or did not define elimination and HIV prevalence among MSM by country. Studies which considered multiple countries were counted for each modeled country [29,38,106,124].* No data available for HIV prevalence among MSM in Mozambique or Ethiopia. The study [37] that did not apply to any specific location was not counted. MSM = men who have sex with men. Country boundary data derived from the Natural Earth dataset (https://www.naturalearthdata.com/).

Fig 2 shows the worldwide location of the modeled populations, juxtaposed with HIV prevalence among MSM by country. Of the 135 included studies, ninety-seven (71.85%) focused on MSM in Western and Central Europe and North America, twenty (14.81%) on Asia and the Pacific, nine (6.67%) on Western and Central Africa, four (2.96%) on Latin America, two (1.48%) on Eastern and Southern Africa, two (1.48%) on Eastern Europe and Central Asia and two (1.48%) on two UNAIDS regions [38,106], and one (0.74%) was not associated with a specified geographical location [37] (Fig 2A). No studies meeting the criteria for our review focused on MSM in the Caribbean or the Middle East and North Africa. Almost half of included studies considered MSM populations in the USA (n = 64, 47.41%), with the second and third most frequently considered locations being the Netherlands (n = 13, 9.63%) and China (n = 9, 6.67%) (Fig 2C).

We observed a striking discordance between the geography of the modeled populations and HIV prevalence among MSM in those populations (Fig 2B and 2C).

### Population characteristics and model types

The distribution of studies by the characteristics of the modeled population and model types across UNAIDS regions is shown in Table 2. No studies on populations outside the USA stratified by ethnicity, whereas 34 studies on populations in the USA did stratify by ethnicity. Commonly considered subgroups were non-Hispanic Black or African American, Hispanic or Latino, non-Hispanic White or other (e.g., [25,47,52]). Most studies that included stratification by ethnicity explored the effectiveness of interventions in achieving the dual goals of reducing the overall HIV burden and narrowing racial disparities (e.g., [52,54,63]). None of the studies in other regions used stratification by ethnicity.

**Table 2:** Number of studies by characteristics of the modeled population and model types. MSM = men who have sex with men. DCM = deterministic compartmental model. SCM = stochastic compartmental model. ABM = agent-based model. UNAIDS = The Joint United Nations Programme on HIV/AIDS.

Population characteristics	UNAIDS regions^*^							
		Western and Central Europe	Latin America	Western and	Eastern and	Eastern Europe and	Asia and	Total
		and North America		Central Africa	Southern Africa	Central Asia	The Pacific	
		n (%)	n (%)	n (%)	n (%)	n (%)	n (%)	n (%)
Ethnicity	Yes	34* (34.34)	0 (0.00)	0 (0.00)	0 (0.00)	0 (0.00)	0 (0.00)	**34** (24.65)
	No	65 (65.66)	4 (100.00)	9 (100.00)	3 (100.00)	2 (100.00)	20 (100.00)	104* (75.36)
Age	Yes	52 (52.53)	2 (50.00)	8 (88.89)	2 (66.67)	2 (100.00)	2 (10.00)	**68** (49.28)
	No	47 (47.47)	2 (50.00)	1 (11.11)	1 (33.33)	0 (0.00)	18 (80.00)	70^*^ (50.72)
MSM only	Yes	87 (87.88)	0 (0.00)	1 (11.11)	0 (0.00)	1 (50.00)	16 (80.00)	106* (76.81)
(vs MSM + other subgroups)	No	12 (12.18)	4 (100.00)	8 (88.89)	3 (100.00)	1 (50.00)	4 (20.00)	**32** (23.19)
Geographical scale	National	34 (34.35)	1 (25.00)	4 (44.44)	3 (100.00)	2 (100.00)	11 (55.00)	**55** (39.89)
	Regional	15 (15.15)	0 (0.0)	0 (0.00)	0 (0.00)	0 (0.00)	2 (10.00)	**17** (12.32)
	Urban	50 (50.51)	3 (75.00)	5 (55.56)	0 (0.00)	0 (0.00)	7 (35.00)	**65** (47.10)
Model type	DCM	35 (35.35)	4 (100.00)	8 (88.89)	1 (33.33)	2 (100.00)	15 (75.00)	66^*^ (47.83)
	SCM	9 (9.09)	0 (0.00)	1 (11.11)	1 (33.33)	0 (0.00)	3 (15.00)	**14** (10.14)
	ABM	55 (55.56)	0 (0.00)	0 (0.00)	1 (33.33)	0 (0.00)	2 (10.00)	**58** (42.03)
**Total**		**99** ^†^	**4**	**9**	**3**	**2**	**20**	**138** ^*,†^

^*^The study [37] considered only MSM using a DCM and did not reference any specific location, geographical scale, age, or ethnicity. The study [38] that included both the USA and Thailand was counted for two UNAIDS regions. The study [106] which considered countries across Africa was counted for two UNAIDS regions.

^†^The study [131] considered 3 different models, and so is counted three times in “Western and Central Europe and North America” to account for each model’s population characteristics.

Sixty eight (49.29%) studies stratified MSM by age. The age ranges mostly covered sexually active MSM, spanning from 13–18 years to 60–80 years. Two studies, both in USA populations, included adolescent sexual minority men (13–18 years old) to investigate the impact of interventions on HIV burden in this group specifically [55,63]. The majority of studies considered populations consisting solely of MSM (n = 106, 76.81%) (e.g., [16,27,133,151]). The remaining studies (n = 32, 23.19%) included other subgroups, such as the heterosexual population [18], transgender women [39], injecting drug users, or female sex workers and their clients [111]. Most studies for settings in Western and Central Africa (n = 7, 77.78%) [98,101,102,106,112,124,125], Eastern and Southern Africa (n = 3, 100%) [71,106,133] and Eastern Europe and Central Asia (n = 1,50.00%) [29] considered sexual mixing of MSM and the consequent cross-transmission of HIV with other subgroups. Studies for Western or Asian countries mostly consider MSM as a separate key population with just a few studies considering MSM mixing with other subgroups in Western and Central Europe and North America (n = 12, 12.18%)(e.g., [16,87,149] or Asia and the Pacific (n = 1, 5.00%) [66]. Most studies developed models for an urban environment (n = 65, 47.10%), while the remaining studies developed national (n = 55, 39.89%) and regional (n = 17, 12.32%) models, or did not mention any particular geographical scale (n = 1, 0.72%) [37].

DCMs were the most frequently used model type overall (n = 66, 47.83%), being the main model type in Latin America (n = 4, 100.00%) [28,39,46,137], Western and Central Africa (n = 8, 88.89%) (e.g., [94,102,112]), Eastern Europe and Central Asia (n = 2, 100.00%) [29,136] and Asia and the Pacific (n = 15, 75.00%) (e.g., [50,111,152]). ABMs were the second most used model type (n = 58, 42.03%) and were rarely used outside of Western and Central Europe and North America (n = 3, 5.17%) [38,84,133]. SCMs were rarely used (n = 14, 10.14%).

### Interventions

Studies investigated classical behavioral interventions (partner reduction and condom use), biomedical interventions (PrEP, post-exposure prophylaxis (PEP), test-and-treat, voluntary medical male circumcision (VMMC), and STI treatment), structural, and HIV vaccine/cure interventions.

**Fig 3:**
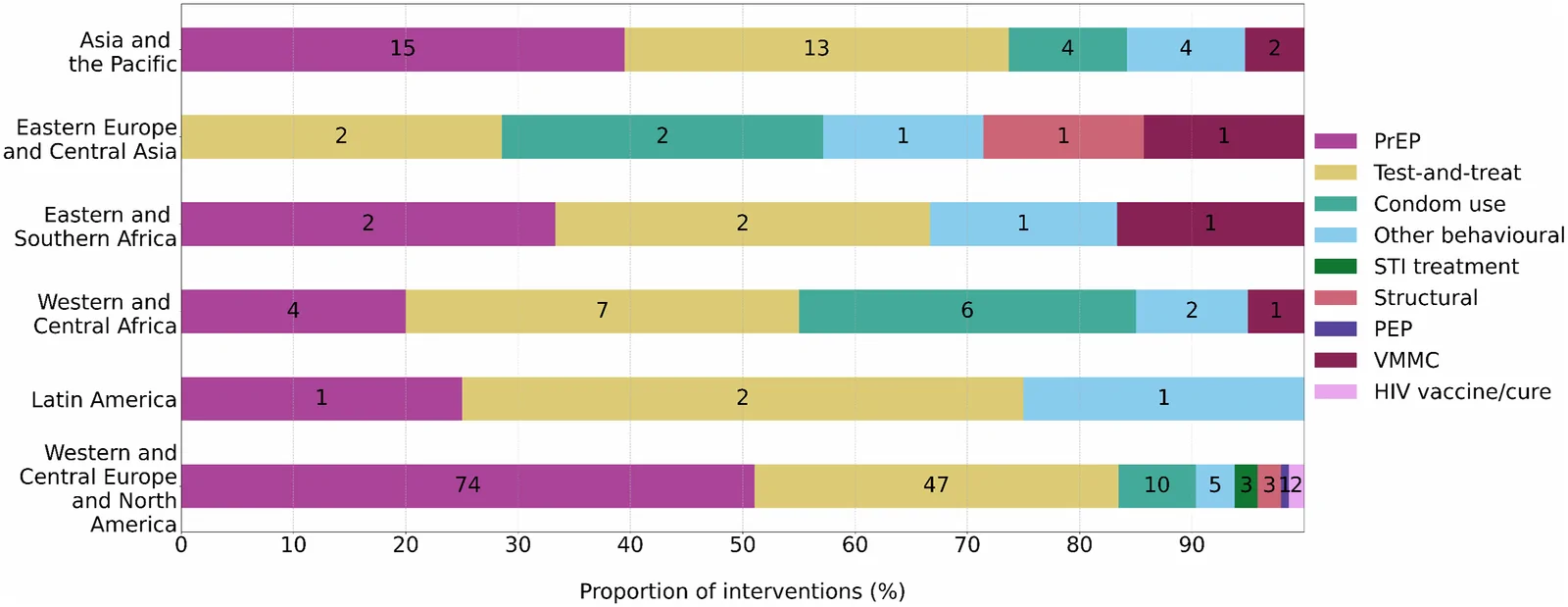
Distribution of modeled interventions by UNAIDS region. The labels on each bar represent the number of times the intervention was included in models. A single study might have included multiple interventions. The study [37] that did not apply to any specific location was not counted. The study [38] that included both the USA and Thailand was counted for two UNAIDS regions. The study [106] which considered countries across Africa was counted for two UNAIDS regions. PrEP = Pre-exposure prophylaxis. STI = sexually transmitted infection. PEP = post-exposure prophylaxis. VMMC = voluntary medical male circumcision. The distribution of interventions by UNAIDS region for studies that defined elimination is shown in S2 Fig in S1 Text.

Classical behavioral interventions facilitated changes in the behavior of MSM pertinent to HIV transmission, such as a reduction in the number of sexual partners (e.g., [97,99,143]), increased use or effectiveness of condoms (e.g., [46,58,101]) and discouraging stimulant use (e.g., methamphetamines, crack/cocaine, and ecstasy) [91]. The term test-and-treat was used to describe interventions that accelerated HIV testing and/or ART coverage (e.g., [37,110,123]). Structural interventions involved changes in healthcare systems or support for MSM at risk, such as providing housing for homeless MSM [105]. Fig 3 shows the distribution of interventions by type and UNAIDS region. Globally, the most frequently studied interventions were biomedical, namely PrEP and test-and-treat, followed by classical behaviour HIV prevention approaches. In contrast, STI treatment, PEP, VMMC, structural and cure/vaccine interventions were rarely included in modeling studies. PrEP was considered more often (n = 74, 51.03%) in Western and Central Europe and North America (e.g., [55,77,81]) than in other regions (n = 1, 25.00% in Latin America [28]; n = 4, 20.00% in Western and Central Africa [94,98,106,112]; n = 2, 33.33% in Eastern and Southern Africa [71,106]; n = 0, 0.00% in Eastern Europe and Central Asia; n = 15, 39.47% in Asia and the Pacific (e.g., [38,111,143])). The next most studied intervention, test-and-treat, was considered frequently across all included regions (n = 47, 32.41% in Western and Central Europe and North America, e.g., [58,87,105]; n = 2, 50.00% in Latin America [46,137]; n = 7, 35.00% in Western and Central Africa, e.g., [101,102,112]; n = 2, 33.33% in Eastern and Southern Africa [106,133]; n = 2, 28.57% in Eastern Europe and Central Asia [29,136]; n = 13, 34.21% in Asia and the Pacific, e.g., [38,96,97].

Classical behavioral interventions were studied frequently outside Western and Central Europe and North America. Combinations of different interventions were more frequently investigated in Eastern Europe and Central Asia (n = 2, 100.00%) [29,136], Western and Central Africa (n = 7, 77.78%) (e.g., [102,112,126]), Asia and the Pacific (n = 14, 70.00%) (e.g., [96,97,143]) and Latin America (n = 2, 50.00%) [28,46] than in Western and Central Europe and North America (n = 35, 36.08%) [58,74,115] or Eastern and Southern Africa (n = 1, 33.33%) [106] (S1 Fig in S1 Text).

### Critical appraisal

Out of the 135 studies investigating the impact of interventions, 41 studies (30.37%) defined criteria for HIV elimination. The critical appraisal results of these 41 studies are summarized in S5 and S6 Tables in S2 Text. The model comprehensiveness scores ranged from 0 to 5, although no study received either of these extreme scores. Studies using ABMs scored at least 2, while three studies (12.50%) using DCMs scored 1.5 or lower [37,59,143], and one study (25.00%) using an SCM scored 1.5 [104]. The average score for ABMs (3.00) was higher than the average scores for DCMs (2.81) and SCMs (2.25). However, three studies (12.50) using DCMs achieved a score of 4.00 [35,93,111], whereas one (7.69%) of the studies using ABMs did [147].

Two of the five criteria, reporting outcomes with uncertainty and accounting for adherence to primary interventions, were well satisfied across all studies. All but three studies (7.32%) [47,104,149] presented results with some level of uncertainty, whether due to sensitivity analyses, stochastic effects, or multiple parameter sets. Only one study 2.44%) [37] did not account for adherence to the primary interventions. In contrast, the other three criteria were often either not included or only partially included. Seventeen studies (41.46%) (e.g., [35,93,110]) attempted to validate their model outputs, although five of these validations were informal or not clearly described [74,90,91,105,125]. Fourteen studies (34.15%) (e.g., [104,125,149]) modeled open MSM populations, with eight studies (19.51%) using compartmental models and six studies (14.63%) using ABMs. Ten studies (24.39%) (e.g., [49,57,97]) accounted for sexual risk compensation, more often in compartmental models than in ABMs.

### Elimination definitions

Table 3 provides an overview of the 41 studies that defined HIV elimination. There was no consensus on a single definition of elimination. Ten of the 41 (24.39%) studies used multiple definitions [18,32,57,74,93,105,108,128,138,148]. For these studies, we described the achievability of the scenario in the model and reported the feasibility of successful elimination scenarios as determined by the original authors.

**Table 3:** The overview of included studies that defined HIV elimination. In total, 41 out of the 135 studies were included. Elimination achievable indicates whether elimination is technically possible in the modeled scenarios. Elimination feasibility indicates the authors’ judgment on whether it is realistic to implement the modeled elimination scenarios in practice. ND indicates that the feasibility of elimination scenarios was not discussed by the authors. A dash in the elimination feasibility column indicates that a discussion about feasibility is not applicable. It is used for studies where elimination is not possible in the model. The elimination scenario with elimination achievable is the minimum required level of studied interventions. If elimination is not achievable, the elimination scenario describes the level of interventions that came closest to meeting the elimination criteria. Feasibility bottleneck describes the challenges associated with elimination scenarios that are identified as technically achievable but not feasible, as described by the authors. MSM = men who have sex with men. TW = transgender women. PWID = people who inject drugs. FSW = female sex workers. SCM = stochastic compartmental model. DCM = deterministic compartmental model. ABM = agent-based model. ART = antiretroviral treatment. PrEP = pre-exposure prophylaxis. ND = not discussed. - = not applicable.

Authors (Year)	Setting	Model type	Definition of elimination	Elimination achievable	Elimination feasibility	Interventions	Starting year and duration	Elimination scenario	Feasibility bottleneck
**Unspecified setting**									
David et al (2020) [37]	Gay, bisexual and other MSM; unspecified setting	DCM	Reproduction number <1	Yes	Unlikely	Test-and-treat	Steady state	Decrease in ART initiation time to less than 1 week	ND
**Asia and the Pacific**									
**China**									
Li et al (2018) [92]	General MSM; China	DCM	<1 new infection per 1,000 person-years	Yes	Likely^j^	PrEP + test-and-treat	2018 20 years	Introducing PrEP coverage to 50% of high-risk MSM + increasing test-and-treat cascade from 68-67-91–90-90-90 reaches elimination by 2037 or increasing PrEP coverage to 75% of high-risk MSM + increasing test-and-treat cascade from 68-67-91–90-90-90 reaches elimination by 2033	–
Lou et al (2017) [97]	General MSM; Beijing, China	DCM	Reproduction number <1	Yes	ND	Test-and-treat + condom use + other behavioral	Steady state	Increase of ART coverage + increase of condom use and/or substantial reduction in the number of sexual partners^*a*^	–
Lu et al (2020) [99]	General MSM; Zhejiang province, China	DCM	Reproduction number <1	No	–	Test-and-treat	Steady state	Increase of test-and-treat parameters to reach 90-90-90 targets	–
Wang et al (2018) [145]	General MSM; China	DCM	<1 new infection per 1,000 person-years	Yes	ND	PrEP + test-and-treat	2018 20 years	Introduce PrEP with 50% coverage in high-risk MSM + increase test-and-treat parameters to reach 90-90-90 targets	–
**India**									
Mitchell et al (2016) [111]	MSM + FSW + clients + general population; Bangalore, India	DCM	<1 new infection per 1,000 person-years	Yes	Likely	PrEP	2017 20 years	Introduction of PrEP among MSM & FSW progressively increasing to 60% coverage within five years, subsequently maintaining this level for the remainder of simulated time, combined with 50% adherence	–
**Japan**									
Gilmour et al (2020) [49]	General MSM; Japan	DCM	<1 new infection per 1,000 person-years	Yes	Likely	PrEP + test-and-treat	2020 30 years	Introduction of PrEP at 25% coverage among high-risk MSM achieved instantaneously at the time of intervention start + test-and-treat reaching 95-95-95 targets immediately	–
Wang et al (2022) [143]	General MSM; Japan	DCM	<1 new infection per 1,000 person-years	Yes	Likely	PrEP + test-and-treat + condom use + other behavioral	2010 40 years	Introduction of PrEP at 10% coverage and 90% effectiveness or increasing annual testing rate and ART coverage to above 80% among all MSM/diagnosed MSM, respectively or condom use increasing from 35% to 65% or high-risk MSM reducing annual partner numbers to less than 9	–
**Taiwan**									
Wu et al (2025) [148]	General MSM; Taiwan	SCM	i) Reproduction number < 1 ii) < 1 new infection per 1,000 person-years	i) Yes ii) Yes	i) Likely^*j*^ ii) Likely^*j*^	PrEP + test-and-treat	2021 30 years	Introducing PrEP to high-risk MSM aged 15–44 with 50% coverage or introducing PrEP to high-risk MSM aged 15–44 with 25% coverage + increasing annual testing to 90% coverage with immediate ART implementation	–
**Western and Central Africa**									
**Cameroon**									
Silhol et al (2021a) [125]	MSM + FSW + clients + general population; Yaoundé, Cameroon	DCM	< 1 new infection per 1,000 person-years by 2030	No	–	Test-and-treat + condom use	2018 12 years	Increase in viral suppression rate from 85% to 90% by 2030 with a 10% ART drop-out rate + condom use increasing from 33% in 2011 to 85% for MSM and their male partners^*b*^	–
**Co&tcirc;e d’Ivoire**									
Maheu-Giroux et al (2017a) [101]	MSM + FSW + clients + general population; Côte d’Ivoire	DCM	< 1 new infection per 1,000 person-years by 2030	Yes	ND	Test-and-treat + condom use	2015 15 years	The threshold will be reached without additional interventions. The threshold can be reached most quickly by achieving 90-90-90 targets by 2020 + 95-95-95 targets by 2030 + 95% condom use among MSM and FSW by 2020	–
**Western and Central Europe and North America**									
**Canada**									
Lima et al (2021) [93]	General MSM; British Columbia, Canada	DCM	i) Reproduction number < 1 ii) < 1 new infection per 1,000 person-years^*c*^	i) Yes ii) Yes	i) ND ii) ND	PrEP + test-and-treat	2019 10 years	Increasing PrEP coverage, adherence, and retention among high-risk/young MSM by 20% + increasing test-and-treat parameters (diagnosis rate, treatment initiation rate, time suppressed and re-initiation rate) by 20% or only increasing test-and-treat parameters by 40%	–
Milwid et al (2022) [107]	Gay and bisexual MSM; Montreal, Canada	ABM	Zero new infections by 2030	-^*d*^	–	PrEP + test-and-treat + condom use + PEP	2000 20 years	2013 onwards: annual PrEP uptake of 6%-32% among eligible MSM + 68.8% - 78.9% proportion of MSM tested annually + 30.0% - 42.3% condom use probability + 2001 onwards: PEP among 0.03%-0.11% of eligible MSM	–
**Denmark**									
Palk et al (2018) [4]	General MSM; Denmark	SCM	< 1 new infection per 1,000 person-years	Yes	Likely	PrEP + test-and-treat	2018 12 years	In the baseline without a PrEP program + 80% ART coverage in people with HIV, elimination is reached by 2030. The introduction of PrEP with coverage in the interval of 0–50% & 60% effectiveness + 3-fold increase in the diagnosis rate will decrease the time to elimination	–
**France**									
Jijon et al (2021) [78]	General MSM; Paris region, France	DCM	Reproduction number < 1	Yes	ND	PrEP + test-and-treat	Steady state	Increase of PrEP coverage from 47% to 55% among high-risk MSM + HIV testing increasing from every 3 years to 4 times a year for those on PrEP	–
**Netherlands**									
Rozhnova et al (2018) [16]	General MSM; The Netherlands	DCM	Reproduction number <1	Yes	Likely	PrEP + test-and-treat	Steady state	82% PrEP coverage (86% effectiveness) among high-risk MSM or 70% PrEP coverage among high-risk MSM + ART coverage in people with HIV increase from 80% to 89%	–
Rozhnova et al (2016) [123]	General MSM; The Netherlands	DCM	Reproduction number < 1	Yes	Unlikely	Test-and-treat	Steady state	No interventions required for fully proportionate mixing between risk groups. For slightly heterogeneous mixing, 30% annual ART uptake (80% coverage of MSM with HIV). For highly heterogeneous mixing elimination is not possible	Elimination is possible when sexual mixing is not realistic, but high and unrealistic levels of ART uptake are required when mixing is realistic.
Wang et al (2025) [142]	General MSM; The Netherlands	DCM	Zero incidence by 2030	Yes	ND	PrEP	2022 8 years	Expanding PrEP availability from 2022 so that two-thirds of eligible/intending MSM use PrEP by 2024	–
**Sweden**									
Hansson et al (2020) [64]	General MSM; Sweden	DCM	Zero endemic prevalence	Yes	Unlikely	PrEP	Steady state	3.5% PrEP coverage among all MSM targeted at high-risk MSM (33.7% of all MSM) or 34.4% PrEP coverage among all MSM targeted at low-risk MSM (66.3% of all MSM)	Using realistic levels of PrEP coverage results in elimination occuring only in the very long-term (more than 50 years). Unrealistic levels of PrEP coverage required for short-term elimination.
Hansson et al (2019) [65]	General MSM; Stockholm, Sweden	SCM	Reproduction number < 1	Yes	Likely	Test-and-treat + condom use	Steady state	Reduction of the time to diagnosis and treatment from 2.86 to < 2.5 years for all partnerships or increase in condom use from 52% to 69% in steady partnerships or from 63% to 72% in casual partnerships	–
**UK**									
Cambiano et al (2023) [32]	Gay and bisexual MSM; UK	ABM	i) < 250 new infections by 2025 ii) < 50 new infections by 2030	i) Yes ii) Yes	i) Unlikely ii) Unlikely	PrEP + test-and-treat	2023 80 years	Increase in PrEP coverage among eligible MSM from approximately 70,000 at peak to 140,000 + 30% increase in HIV testing rate	Cost-effective levels of PrEP coverage make achieving elimination goals, particularly goal ii, unlikely. Costs of PrEP and testing need to decrease to allow for higher and still cost-effective coverage.
Massey et al (2023) [104]	General MSM; UK	SCM	60% incidence reduction by 2030	Yes	Unlikely	PrEP + test-and-treat	2020 30 years	Increase in viral suppression among those treated from 97% to 99% + either increase in the annual probability of screening from 22.35% to 96.85% or increase in annual PrEP uptake from 0.08% to 9.08%	Large and unfeasible increases in PrEP usage and screening rates required.
**USA**									
Chen et al (2022) [35]	African American/ Hispanic/ Other MSM + PWID + heterosexual population; USA	DCM	Reproduction number substantially $<1^{e}$	Yes	Unlikely	Test-and-treat	2017 3 years	50% reduction of viral suppression loss rate	Results indicate effective reproduction number only slightly below 1, so elimination may not happen or will at least take a very long time. Strengthened and ongoing efforts to diagnose HIV infection early, link the diagnosed to care, and help those receiving treatment to sustain viral suppression all required for increased likelihood of elimination.
Fojo et al (2021) [45]	African American/ Hispanic/ Other MSM + heterosexual population + PWID; 32 urban areas of highest HIV burden and Washington DC, USA	DCM	90% incidence reduction by 2030^*f*^	Yes	Unlikely	PrEP + test-and-treat	2020 10 years	25% PrEP coverage among those eligible + 90% viral suppression among diagnosed + twice-yearly testing	Higher levels of PrEP availability and test-and-treat rates can lead to reaching the goals but would require substantially more resources than feasible.
Fraysse et al (2025) [47]	General MSM; Atlanta, Georgia, USA	ABM	25% incidence reduction by 2030 compared to unclear baseline	Yes	Likely	PrEP	2023 7 years	Increasing PrEP coverage from 14.3% to 28.9%-42.2% depending on relative use of long-acting PrEP or daily PrEP, respectively	–
Le Guillou et al (2021) [57]	African American/ Hispanic/ White MSM; San Francisco, USA	ABM	i) 75% incidence reduction by 2025 + ii) 90% reduction by 2030^*f*^	i) Yes ii) No	i) Unlikely ii) -	PrEP + test-and-treat	2019 10 years	Increase in PrEP coverage from 25% to 75% + 60% viral suppression among all MSM with HIV or from 25% to 62.5% + 68% viral suppression among all MSM with HIV	Scale up of PrEP and ART required to achieve elimination goals by 2025 is too large to be feasible.
Gutowska et al (2022) [59]	General MSM; USA	DCM	Reproduction number <1	Yes^*g*^	Unlikely	PrEP	Steady state	Model with only short-term partnerships: no PrEP needed; model with only long-term partnerships: 40% PrEP coverage and 95% adherence; model with both types of partnerships: PrEP does not lead to elimination	Unrealistic levels of PrEP coverage and adherence are required for elimination.
Hamilton et al (2023) [18]	African American/ Hispanic/ Other MSM + heterosexual population; 16 states and District of Columbia in the South census region, USA	ABM	i) 75% incidence reduction by 2025 + ii) 90% reduction by 2030^*f*^	i) Yes ii) Yes	i) ND ii) ND	PrEP + test-and-treat	2022 8 years	PrEP coverage among eligible + ART coverage among all people with HIV of i) 40% and 90% or ii) 50% and 100%	–
Jenness et al (2020) [74]	African American/ Hispanic/ White MSM; Atlanta, Georgia, USA	ABM	i) 75% incidence reduction by 2025 + ii) 90% reduction by 2030^*f*^	i) Yes ii) Yes^*h*^	i) ND ii) ND	PrEP + test-and-treat	2020 10 years	PrEP linkage for eligible MSM via screening resulting in a PrEP coverage increase from 15% to 67% + 10-fold increase in HIV screening and care retention resulting in quarterly screening for white MSM & twice-yearly for African American/Hispanic MSM	–
Khanna et al (2021) [85]	Young African American MSM; State of Illinois, USA	ABM	<200 new infections per year by 2030^*i*^	No	–	PrEP + test-and-treat	2016 15 years	Increase in PrEP coverage from 10% to 40% + increase in ART coverage among all MSM with HIV from 50% to 80%	–
Khanna et al (2019) [86]	Young African American MSM; State of Illinois, USA	ABM	<200 new infections per year by 2030^*i*^	Yes	Unlikely	PrEP	2020 10 years	Increased PrEP coverage from 13% to 50% prioritizing individuals in serodiscordant partnerships	Scaling PrEP coverage to this degree is unrealistic. Therefore consider combination interventions with ART and psychosocial/structural barriers to treatment.
Lee et al (2023) [90]	Young African American MSM; Houston, Texas, USA	ABM	<200 new infections per year by 2030^*i*^	No	–	PrEP + test-and-treat	2020 10 years	Increase in PrEP coverage from 19.6% to 49.6% + increase in ART coverage from 63% to 78% + 30% reduction in the proportion of individuals who tested 1–2 times in the last two years from 42.7% to 12.7% + increase in the proportion of individuals who initiate treatment within 1 week of diagnosis from 3.8% to 63.8%	
Lee et al (2022) [91]	Young African American MSM; State of Illinois, USA	ABM	<200 new infections per year by 2030*^i^*	Yes	ND	PrEP + test-and-treat + other behavioral	2020 10 years	30% increase in PrEP coverage from 13.7% + 30% increase of ART coverage + 25% coverage of behavioral interventions aiming to reduce stimulant use coverage among stimulant users	–
Martin et al (2020) [105]	MSM + general population; New York State, USA	DCM	By the end of 2020: i) < 750 new infections per year ii) achieve a decrease in annual HIV prevalence iii) reduce AIDS progression	i) Yes*^h^* ii) Yes iii) Yes	i) Unlikely ii) Unlikely iii) Unlikely	PrEP + test-and-treat + structural	2014 12 years	Introduction of PrEP coverage at 25–75% with 50–100% adherence among MSM + increased test-and-treat (25–75% increase in ART uptake & 85–135% decrease in time between ART initiation and viral suppression) + housing assistance (0–10% reduction in HIV infectivity & 15–55% increase in ART initiation rate and 15–55% decrease in time between ART initiation and viral suppression)	Unrealistic intervention scenarios required to be likely to achieve elimination by 2020.
Mitchell et al (2023) [108]	African American/ White MSM; Atlanta, Georgia, USA	DCM	i) 75% incidence reduction by 2025 + ii) 90% reduction by 2030^*f*^	i) Yes ii) Yes	i) Unlikely ii) Unlikely	PrEP	2022 8 years	i) 97% coverage of oral PrEP or 73% coverage of injectable PrEP ii) 100% coverage of oral PrEP is insufficient but 93% coverage of injectible PrEP (including ≥90% of PrEP-indicated MSM) is sufficient	Very high and therefore unrealistic PrEP coverage required for elimination. Long-acting injectable PrEP is found to contribute substantially in lower coverages, so combination interventions should be considered.
Mitchell et al (2019) [110]	African American/White MSM; Baltimore, Maryland, USA	DCM	<1 new infection per 1,000 person-years	Yes	Unlikely	Test-and-treat	2016 20 years	Increase in ART coverage among those diagnosed to 86% from 66% after five years resulting in a 50% relative reduction in HIV incidence rate leads to the highest probability (5.9%) of achieving elimination by 2036	Very unlikely to achieve elimination by 2036 with the modelled interventions. Elimination can be achieved later than 2036 with these interventions, or possibly by 2036 if considering further interventions such as PrEP.
Singleton et al (2020) [128]	African American/ White MSM; Atlanta, Georgia, USA	ABM	i) 90% incidence reduction by 2030f + ii) <1 new infection per 1,000 person-years	i) No ii) No	i) - ii) -	PrEP + test-and-treat	2015 10 years	i) & ii) 90% PrEP coverage among all eligible MSM + expanding test-and-treat to achieve 95-95-95 targets iii) Eliminating PrEP adherence disparities by increasing the proportion of African American MSM on PrEP who take four or more doses per week from 56.8% to 91.1%, the same as White MSM	–
Tollet et al (2024) [135]	General MSM; Arizona, USA	DCM	Reproduction number < 1	Yes	ND	PrEP + test-and-treat + other behavioural	Steady state	Increase in PrEP coverage among eligble MSM from 31% to 47–76% or infected individuals on treatment within 6 years of acquiring HIV or behaviour change in high-risk individuals so that they are at most 15% more likely to acquire HIV than low-risk individuals	–
Vermeer et al (2022) [138]	African American/ Hispanic/ White MSM; Chicago, Illinois, USA	ABM	i) 75% incidence reduction by 2025 + ii) 90% reduction by 2030^*f*^	i) Yes ii) Yes	i) Unlikely ii) Unlikely	PrEP + test-and-treat	2015 15 years	i) PrEP linkage (proportion of MSM who initiate PrEP after a negative test) increase from 7% to 47% + 75% annual PrEP retention + 100% PrEP adherence, succeeding in 77% of simulations ii) PrEP linkage increase from 7% to 67% + 75% annual PrEP retention + 100% PrEP adherence + increase in ART retention to 99%, succeeding in 58% of simulations	Considerable scaling up of interventions and strategies in both the prevention and care cascades are required, with PrEP playing a central role.
Wheatly et al (2022) [147]	General MSM; Atlabta, Georgia, USA	ABM	90% incidence reduction by 2030^*f*^	No	–	PrEP	2020 20 years	25% of MSM increasing PrEP initiation probability by factor of 4, 30% of MSM increasing PrEP adherence from 60% to 87.1%, 40% of MSM increasing PrEP retention from median of 227 days to median of 347 days	–
Zang et al (2023) [149]	African American/ Hispanic/ White MSM + PWID + general population; Miami-Dade Country, Florida, USA	DCM	i) 90% incidence reduction by 2030^*f*^	No	–	PrEP + test-and-treat	2022 8 years	Scale-up proportionally across ethnic groups until and beyond 2026: Testing-rate increase of 26% + PrEP initation rate increase 212.5% + proportion of rapid (30-day) ART initiation increase 19.9% + ART re-engagement rate increase of 111% + decrease ART dropout to 27% of baseline	–

^*a*^ Model parameters and the effect of ART coverage are not clearly described in the model.

^*b*^ Model uses different values for partnerships between all population subgroups. Male-male partnerships are given as an example.

^*c*^ WHO incidence threshold of <1 new infection per 1,000 person-years.

^*d*^ The study did not assess elimination prospects but reconstructed the past pandemic to guide future elimination efforts.

^*e*^ Reproduction number substantially <1 is not clearly defined, the baseline reproduction number is already <1.

^*f*^ Ending the HIV epidemic goals in the USA/Getting to zero goals in the state of Illinois, USA.

^*g*^ Elimination prospects depend on the assumptions about sexual behavior.

^*h*^ Elimination is achieved, but not by the target time.

^*i*^ Functional zero target, i.e., a level of new annual infections at which the epidemic in the USA cannot be sustained.

^*j*^ Likely as study authors deem required intervention scenario to be cost-effective.

HIV elimination criteria were defined as a threshold for incidence (n = 19, 36.54%; e.g., < 1 new infection per 1,000 person-years [4,110,143]), a percentage reduction in incidence (n = 16, 30.77%; e.g., 90% reduction by 2030 from 2020 [45,57,108]), reproduction number less than one (n = 12, 23.08%; e,g., [37,59,99]), zero incidence (n = 2, 3.85% [107,142]), zero steady-state prevalence (n = 1, 1.92% [64]), and other (n = 2, 3.85% [128]) Six studies in the USA investigated scenarios where elimination was, at least in part, defined as eliminating disparities between ethnicities. We decided not to consider these as elimination scenarios in our review as they reflect a different aim.

**Fig 4:**
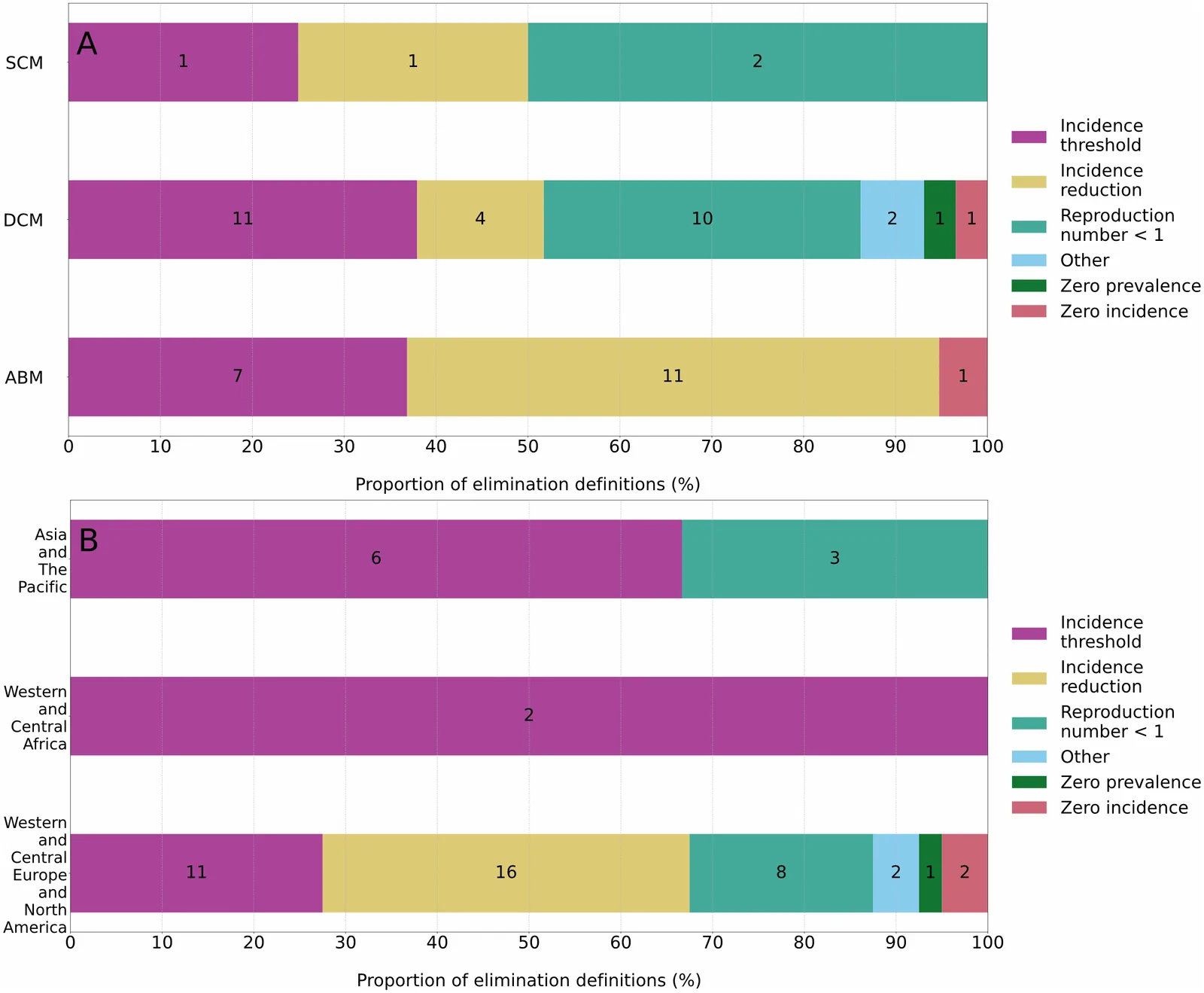
HIV elimination definitions. Distribution of definitions by (A) model type and **(B)** UNAIDS region. The labels on each bar represent the number of times the definition was included in studies. A single study might include multiple definitions. In **(B)**, the study [37] that did not reference any specific location was not counted. SCM = stochastic compartmental model. DCM = deterministic compartmental model. ABM = agent-based model.

Elimination definitions varied across model types and UNAIDS regions (Fig 4). A reproduction number less than one was the most frequently used definition in compartmental models (n = 12, 36.36%) (e.g., [35,37,59]) but was absent in ABMs. Conversely, incidence reduction was the most frequent definition in ABMs (n = 11, 57.89%) (e.g., [18,57,74]) but was seldom used in compartmental models (n = 5, 15.15%) [45,104,108]. Incidence threshold criteria were used across all model types (n = 19, 36.54%) (e.g., [4,90,110]).

The preferential use of elimination definitions by UNAIDS region (Fig 4B) resulted mainly from (i) ABMs not being used for settings outside Western and Central Europe and North America, specifically the USA, and (ii) studies often aligning their elimination definitions with the national goals of the country where elimination was assessed. For example, incidence reduction accounted for over half of the definitions used in the USA (n = 15, 55.56%) (e.g., [45,74,128]), driven by the goals to end the HIV epidemic in the USA, which aim for 75% and 90% reductions in incidence by 2025 and 2030, respectively. In contrast, studies on populations outside of the USA may have different or no national goals to guide them. Consequently, these studies focused on reaching incidence thresholds (e.g., [49,93,125]) and reducing the reproduction number below one (e.g., [97,99,123]), and did not use incidence reduction as the elimination definition.

We observed that elimination scenarios were not studied uniformly across the review inclusion period (2016–2025). The total number of published elimination scenarios peaked at 11 in 2020, rising from 2 in 2016 and declining again from 2024 (S3 Fig in S1 Text). Scenarios were described as not being achievable only during the peak publishing period of 2020–2023.

### Elimination prospects

In the following, we distinguished between elimination being (i) achievable, if it was technically possible within modeled scenarios, and (ii) feasible, if, based on the authors’ judgment and discussion, the modeled scenarios where elimination was achievable were practical in a real-world context (Table 4). Feasibility was considered (un)likely if the authors judged that the intervention scenario required to achieve elimination in the model was (im)plausible. In 42 out of 52 modeled scenarios (80.77%), elimination was achievable. Using UNAIDS regional stratification, thirty-two (82.05%) scenarios in Western and Central Europe and North America (e.g., [16,86,110]), one (50.00%) scenario in Western and Central Africa [102], and eight (88.89%) scenarios in Asia and the Pacific (e.g., [97,111,143]) could achieve elimination in the model. The feasibility of elimination was discussed in 28 (66.67%) of the 42 modeled scenarios. However, only 10 35.71%) of these 28 scenarios were deemed likely to be feasible in practice, 4 (40.00%) of which were in Western and Central Europe and North America, e.g.,([4,16,65]) and 6 (60.00%) in Asia and the Pacific (e.g., [49,111,143]). In eighteen (64.29%) elimination scenarios where feasibility was discussed, elimination was deemed unlikely to be feasible in practice, all for settings in Western and Central Europe and North America (e.g., [64,105,123]) or without a specific location [37]. The feasibility of fourteen of forty-two (33.33%) scenarios was not discussed by the authors (e.g., [18,91,102]).

**Table 4:** Elimination prospects. MSM = men who have sex with men. SCM = stochastic compartmental model. DCM = deterministic compartmental model. ABM = agent-based model. UNAIDS = The Joint United Nations Programme on HIV/AIDS. ND = not discussed.

UNAIDS regions^*^					
		Western and Central Europe	Western and	Asia and	Total
		and North America	Central Africa	The Pacific	
		n (%)	n (%)	n (%)	n (%)
Elimination definition	Incidence threshold	11 (28.21)	2 (100.00)	6 (66.67)	**19** (37.25)
	Incidence reduction	16 (41.03)	0 (0.00)	0 (0.00)	**16** (31.37)
	Reproduction number <1	8 (20.51)	0 (0.00)	3 (33.33)	12^*^ (23.53)
	Other^†^	2 (5.13)	0 (0.00)	0 (0.00)	**2** (3.92)
	Zero prevalence	1 (2.56)	0 (0.00)	0 (0.00)	**1** (1.96)
	Zero incidence	1 (2.56)	0 (0.00)	0 (0.00)	**1** (1.96)
Interventions	Individual	12 (30.77)	0 (0.00)	2 (22.22)	15^*^ (29.41)
	Combination	27 (69.23)	2 (100.00)	7 (77.78)	**36** (70.59)
Elimination achievable	Yes	32 (82.05)	1 (50.00)	8 (88.89)	42^*^ (82.35)
in modeled scenarios	No	7 (17.95)	1 (50.00)	1 (11.11)	**9** (17.65)
**Total**		**39**	**2**	**9**	**51** ^*,‡^
UNAIDS regions^*^					
Elimination feasibility	Likely	4 (12.50)	0 (0.00)	6 (75.00)	**10** (23.81)
if elimination is achievable	Unlikely	17 (53.13)	0 (0.00)	0 (0.00)	18^*^ (42.86)
in modeled scenarios	ND	11 (34.38)	1 (100.00)	2 (25.00)	**14** (33.33)
**Total**		**32**	**1**	**8**	**42** ^*^

^*^The study [37] did not reference a specific location and, therefore, was added directly to the totals. In this study, elimination was achieved in the model using test-and-treat and a reproduction number definition, but considered unlikely.

^†^The study [105] used elimination definitions that did not fit into other categories, such as (i) achieving a reduction in annual HIV prevalence by 2020 and (ii) reducing AIDS progression by 2020.

^‡^The study [107] defined elimination as zero HIV incidence among MSM but did not directly assess it; therefore, it was not counted in this table.

Achievability of elimination differed with respect to interventions by UNAIDS region and country (S2 and S3 Tables in S1 Text). The majority of scenarios in Western and Central Europe and North America that included PrEP and/or test-and-treat achieved elimination (n = 26, 81.25%, e.g., [4,59,78] and n = 25, 83.33%, e.g., [18,57,93], respectively), while classical behavioral and structural interventions were included infrequently but always achieved elimination (n = 4, 100.00%, e.g., [65,91,135] and n = 3, 100.00%, e.g., [105], respectively). However, only four elimination scenarios were discussed by the original authors to be feasible, mostly for models set in Europe and considering MSM a closed population [4,16,65], with only one study considering a feasible elimination scenario for MSM in the USA [47]. Elimination scenarios deemed feasible by the authors included an increase of ART coverage and introduction of oral PrEP in the Netherlands [16] and Denmark [4], and a further reduction in time to diagnosis with an increase in condom use in Sweden [65]. In the USA, elimination using a similar composition of interventions was considered unlikely by the authors despite the majority of modeled scenarios predicting elimination (e.g., [35,45,59]) with the notable exception of one study [47] concluding that PrEP can help achieve elimination only if introduced using the long-acting injectible form. In Western and Central Africa, test-and-treat and condom use could achieve elimination in half of the modeled scenarios [102], but their feasibility was not discussed by the authors. In Asia and the Pacific, modeled elimination scenarios included test-and-treat (n = 7, 38.89%), PrEP (n = 7, 38.89%), condom use (n = 2, 11.11%), and other behavioral (n = 2, 11.11%) interventions. Except one scenario that involved test-and-treat only [99], all of them were achievable, and their feasibility was also considered as high by the authors for India [111], Japan [49,143] and Taiwan [148] alongside one (20.00%) elimination scenario in China [92]. The feasible elimination scenarios involved the introduction of oral PrEP and an increase in test-and-treat rates, which could be complemented with condom use and other behavioral interventions. Notably, worldwide, of the ten feasible elimination scenarios, eight (80.00%) used a combination of interventions [4,16,49,65,92,143,147,148], while only one used PrEP as an individual intervention [111]. Elimination prospects stratified by the use of combination and individual interventions are shown in S1 Table in S1 Text.

Looking closer at the scenarios deemed feasible by the authors, the epidemiological context of HIV in MSM across the Netherlands, Denmark and Sweden is similar. Across all three countries, HIV incidence among MSM has been declining in recent years due to a combination of PrEP, early treatment initiation (TasP) and frequent testing [6,154,155], with Sweden achieving 2021 UNAIDS 95-95-95 goals in 2022 [154].

The studies in the Netherlands [16] and Denmark [4] both predict scenarios deemed feasible by the authors through introducing PrEP in addition to increasing ART coverage or decreasing the time to diagnosis. The study in Sweden [65] is the only (10.00%) study which considers elimination feasible without PrEP, rather, in this case, the authors highlight the importance of reducing the time to diagnosis for achieving elimination, alongside increasing the proportion of MSM using condoms. Despite twenty out of thirty-two (62.50%) achievable elimination scenarios in Western and Central Europe and North America being in the USA, only one [47] (5.00%) of these was deemed feasible by the authors. In the USA, eliminating HIV among MSM faces diverse obstacles such as sub-optimal access to medical care or financial assistance, stigma and awareness of PrEP alongside racial disparity barriers [156]. The authors of the study in the USA [47] found elimination feasible in the city of Atlanta, Georgia, through increasing coverage of and switching users to long-acting injectible PrEP, because if solely oral PrEP is considered, the coverage levels need to be too high for elimination to be feasible. However, another study in this review [108] also investigated HIV elimination among MSM by using long-acting injectible PrEP in the same demographic context (Atlanta, Georgia), the authors of this study found oral PrEP coverage as high as 97% or long-acting injectible PrEP coverage of 73% to be required for achieving elimination. These two studies highlight the importance of how elimination is defined. In the first case, [47] elimination is defined as a 25% reduction in HIV incidence by 2030 compared to 2020, and in the other case [108] elimination is defined as a 75% reduction in HIV incidence by 2030 compared to 2020. Both studies are able to satisfy the lower threshold of these two definitions, but neither can achieve a 75% reduction without levels of (long-acting) PrEP coverage deemed infeasible by the authors, leading to differing conclusions on achieving elimination despite achieving similar goals in absolute terms.

In contrast, in Asia and the Pacific, six out of eight (75.00%) successful elimination scenarios were deemed feasible by the authors. Feasible scenarios across Asia were modeled in China [92], India [111], Japan [49,143] and Taiwan [148]. These scenarios all investigate introducing PrEP in addition to a baseline without any PrEP coverage. All but one of these scenarios, in India [111], additionally combined PrEP with test-and-treat interventions such as increased testing or ART coverage. This study introduces PrEP to both MSM and female sex workers, maintaining a high coverage of 60% in these populations for 15 years. In India, access to ART is provided free by the government [157] yet, as of 2015, only 67% of people living with HIV in India were diagnosed and of those diagnosed only 66% were receiving ART [158], suggesting that test-and-treat interventions should also be scaled up alongside PrEP. Broadly speaking, PrEP coverage among MSM across Asia is low, but if barriers to accessing PrEP such as cost can be overcome, then PrEP uptake could increase by over 50% among MSM in Asia [159]. One of the studies in Japan [143] introduces PrEP at 10% coverage among all MSM and increases ART coverage to 80% among diagnosed MSM, while the other study in Japan [49] introduces PrEP at 25% coverage among only MSM with high-risk of HIV acquisition alongside reaching 95-95-95 test-and-treat targets. Achieving high coverages of interventions among MSM with high-risk of HIV acquisition could be easier, due to their tendency to be more connected to sexual health services. The study in China [92] introduces PrEP to 50%-75% of MSM with high-risk of HIV acquisition alongside reaching 90-90-90 test and treat targets. Finally, the study in Taiwan [148] introduces PrEP to 15–44 year old high-risk MSM with 25%-50% coverage alongside 90% annual testing rate and immediate ART implementation.

Thirteen of 14 studies that described elimination feasibility as unlikely also described specific bottleneck’s to achieve elimination (Table 3). The most frequently cited reason (by 11 studies, 78.57%) [32,45,57,59,64,86,104,105,108,123,138] was achieving elimination would require intervention coverage levels that are not feasible, as determined either by the authors’ judgment or by cost-effectiveness analysis. Additional bottlenecks identified by the original authors included the need to strengthen the entire HIV treatment cascade through earlier diagnosis, improved linkage to care, and sustained viral suppression [35]; and the incorporation of expensive additional interventions such as PrEP [110].

## Discussion

### Underrepresented populations

HIV among MSM is a global problem that transcends geographical borders [1]. To our knowledge, this study is the first to systematically review mathematical modeling studies that assess HIV elimination prospects in this key population worldwide. Our findings show that across all UNAIDS regions, many countries with high HIV burden among MSM were not represented in the recent studies that involve dynamic transmission models. In particular, we did not identify any studies that assess the epidemiological impacts of interventions on MSM in the Caribbean or the Middle East and North Africa. Several factors could contribute to this gap in knowledge, including the lack of high quality sexual behavior and epidemiological data needed for model parameterization, potentially shown by the lack of data for the Middle East in Fig 2B, the shortage of local expertise in HIV modeling [160], criminalization of HIV and homosexuality, insufficient interest from public health systems in countries where the HIV epidemic in non-MSM populations is more severe than among MSM, poor surveillance, underfunding, discrimination, and stigma.

Notably, a relatively small number of studies targeted and reported outcomes for MSM in the UNAIDS regions of Western and Central Africa, and Eastern and Southern Africa, collectively known as sub-Saharan Africa [71,94,98,101,102,106,112,124–126,133], where modeling HIV transmission in the general population has traditionally received a lot of attention [161,162].

This region is known for generalized heterosexual epidemics and a high HIV burden in the general population, but there is also strong evidence of epidemics among MSM [10,163,164]. According to the recent estimates [3], in sub-Saharan Africa numbers of new infections in the overall adult population, sex workers and their clients have been falling at the same rate, but no such progress has been observed for MSM, who are left behind.

Some studies which were not included in this review primarily modeled populations other than MSM. A wide range of studies using models such as Optima [165] include MSM as a subpopulation but did not report results specifically. Inclusion and reporting outcomes for MSM in mathematical models for African countries is needed to provide knowledge on tailored interventions that ensure equal rates of progress to HIV elimination for different key populations and the general population [166]. Within the Western and Central Europe and North America UNAIDS region, a relatively small number of studies concerning MSM in Europe were included in our review [4,16,26,32,33,44,56,64,65,70,78,88,104,114,118–120,122,123,141,142,146] compared to the USA. The likely explanation for this is that modeling methods outside the scope of our review are used to investigate HIV elimination among MSM in Europe. For example, back-calculation models have been developed for the Netherlands [6], the UK [5], and Denmark [167] but are not included in our review focused on dynamic transmission models.

### Elimination scenarios

Elimination was achieved in models far more often than authors deemed feasible for real-world implementation (10 out of the 28 scenarios where feasibility was discussed). Several reasons could contribute to this discrepancy. Firstly, intervention parameters in models (e.g., PrEP, ART, condom use coverage and adherence, testing rates) can be selected from the maximum possible range, potentially resulting in values that are not achievable in practice (e.g., [57,105,108]).

Secondly, the feasibility of one-third of modeled elimination scenarios was not discussed by the authors (e.g., [97]), possibly due to the lack of authors with relevant real-world implementation expertise. Thirdly, 9 of the 10 scenarios deemed feasible by the authors [4,16,49,65,92,111,143,148] were obtained in either DCMs or SCMs that were mostly among the least complex models, as described by their comprehensiveness scores. This implies that the results reported in these 9 scenarios may be overly optimistic.

Our findings show that significant gains in HIV control among MSM have been made in some settings. Elimination is likely in certain Western European countries due to the scale-up of test-and-treat programs, and PrEP emerges as one of the key interventions that can help to reach elimination faster [4,47]. This aligns with evidence that HIV incidence in countries like the Netherlands and the UK had started to decline with the expansion of test-and-treat programs [5,6] but declined much further after the introduction of national PrEP programs [168].

Notably, only one of the studies in the USA setting considered elimination feasible. This was in a scenario where PrEP coverage is increased significantly among MSM, and the authors note that this is only achievable with feasible levels of PrEP coverage if using long-acting injectible PrEP rather than a daily pill regimen. This outcome could be partly explained by the complexity of the subepidemics in the USA, characterized by strong heterogeneity in transmission among different ethnic and geographical subgroups that require culturally and regionally tailored interventions.

Our findings also highlight inequitable responses in HIV control worldwide. Unlike many studies in Western countries [4,16,45,47,64,78,91,93,107,108] and some studies in Asia [49,92,111,143,145,148] that consider PrEP as an essential intervention for faster approach to elimination, studies in Africa still focus on the expansion of treatment and condom use [102,125]. However, the real-world effectiveness of condoms is undermined by adherence issues. No studies considered elimination scenarios with PrEP introduction among MSM in Africa, potentially due to delays and structural barriers in implementing this intervention [169].

Our review underscores the importance of combination interventions in increasing the feasibility of elimination. Although the sample of elimination scenarios deemed feasible by the authors was small and their conclusions may be overly optimistic, 8 out of 10 considered a combination of interventions [4,16,49,65,92,143,148]. This is consistent with other studies suggesting that a combination of intervention strategies is necessary to control and eliminate HIV [13,170,171], and that transmission models should incorporate multiple, simultaneously acting interventions [15].

Lastly, a subset of studies in the review aimed to perform a cost-effectiveness analysis of modeled intervention scenarios. Three of the ten elimination scenarios deemed feasible by the authors were in such studies [92,148]. Perhaps the ability of these studies to assess the feasibility of intervention scenarios can be trusted more than studies which do not perform cost-effectiveness analyses as they are investigating how readily these interventions can be implemented in reality.

### Relatedness of elimination definitions

Various definitions of elimination used by different studies reflect their perspectives in terms of modeling and public health. While each elimination definition focuses on a specific aspect of the epidemic dynamics of HIV, these aspects are epidemiologically related. In theory, when the effective reproduction number is below one, the incidence will decrease and eventually elimination will be reached. The smaller the effective reproduction number, the faster the decline in incidence, and the sooner a given incidence threshold will be reached. From a modeling perspective, using the effective reproduction number is attractive (e.g., [65,78,143]), because it summarizes the qualitative behavior of the epidemic, and elimination is a consequence of reducing the reproduction number below the threshold of one. From an estimate of the reproduction number, one could, in principle, compute the incidence and the time it takes for the incidence to fall below a threshold. The converse does not hold, i.e., knowing that the incidence is below a certain threshold does not necessarily mean that elimination will be reached in the long term. The incidence could still stabilize at a new lower endemic prevalence if the reproduction number is above one. Therefore, from a modeling perspective, calculating the effective reproduction number has clear advantages over simply calculating incidence. However, an explicit calculation of the reproduction number is only possible for DCMs which explains the frequent use of this definition for these models in our review (e.g., [16,59,93]). For SCMs and ABMs, approximations for a reproduction number can be computed numerically by calculating the average number of secondary cases per infected individual. The involved numerical computations probably explain why none of the ABMs in our review used this definition.

From a public health perspective, it is essential to consider how the path to elimination can be achieved and monitored [172]. This implies that we need elimination definitions based on measurable quantities [173]. Although incidence is not directly observable, it can be estimated from the number of diagnosed cases. More concretely, definitions based on an epidemiological goal such as a threshold (e.g., [4,85,110]) or a reduction in incidence (e.g., [18,32,45]) can be used in practice to validate model predictions. Both can be measured and monitored over time, and serve as a basis for defining standardized indicators for public health policy evaluation [173]. Therefore, for modeling to contribute effectively to public health policy, it is necessary and valuable to report intervention coverage and incidence, preferably in a format that can easily be compared to standard indicators [172,173]. A clear advantage of using the elimination definition based on an incidence threshold is that incidence can be computed in all model types. Besides offering meaningful guidance to policymakers, this definition would also enhance study comparability within and across different MSM settings.

The elimination of disparities, often discussed in the context of HIV, is a different concept and does not necessarily lead to the elimination of HIV from a population. A few studies (e.g., [52,54,63]) in this review set targets of eliminating racial disparities, either in isolation or alongside other elimination criteria. The goal is to eliminate large differences in HIV incidence between population groups, such as ethnic groups, rather than to eliminate HIV entirely, we therefore decided not to include this definition alongside the others in our review. In many epidemiological contexts, it is an important milestone to overall HIV elimination once we consider that groups with high incidence of risky behavior, are often characterized by a high risk of HIV acquisition, resulting in effective reproduction numbers in these groups remaining above one the longest. In the final stages before overall elimination, the groups with the highest reproduction numbers will be the remaining drivers of transmission. Therefore, targeting these groups will be crucial to achieving the final goal of HIV elimination.

### Future elimination modeling

Based on our findings, we identify several areas where HIV elimination modeling in MSM needs to advance. In countries where HIV incidence has dropped considerably in recent years [4–6] and elimination may be possible, modeling should focus not only on interventions that achieve elimination but also on those that sustain it. This conclusion was also supported by [174]. Elimination of transmission among MSM in specific settings may be interpreted as HIV no longer being a public health problem, which could lead to cutbacks in national prevention programs in these communities. For example, scenarios of future changes in the capacity of the national program for oral PrEP are being investigated in the Netherlands [118]. A decrease in PrEP uptake and condom use, combined with a potential increase in sexual risk behavior among MSM [175–177], may lead to a rebound in HIV incidence. A new priority for countries nearing elimination and aiming to sustain it could be interventions targeting HIV infections acquired abroad, either through immigration or travel. Examples of such interventions include offering voluntary HIV testing to incoming migrants or providing PrEP to MSM who travel abroad [4]. Additionally, transmission modeling will need to be complemented with health economic evaluations to guide future interventions that balance the cost of maintaining them and keeping HIV transmission at bay.

Table 3 showed that the most commonly cited bottleneck to achieving HIV elimination is the requirement for unfeasible levels of intervention engagement (e.g., uptake, retention, coverage, and adherence). We also showed that feasible intervention scenarios most commonly involve combinations of different interventions targeted at both care and prevention. We therefore recommend that future modeling studies focus on head-to-head modeling of combinations of interventions with realistic implementation and intervention engagement, with the goal being to determine which realistic intervention scenarios are best suited to achieving HIV elimination.

Our review highlighted a lack of dynamic modeling studies in some regions that have a high HIV prevalence among MSM. Within locations that warrant such attention, a special place is held by so-called island countries. To wit, the Caribbean region, which has the second highest HIV prevalence among MSM across UNAIDS regions [178] and a high volume of migration both within the region and internationally, will require the development of meta-population models. These models including population mobility were absent in our review. Understanding HIV transmission networks and the impact of migration [179] including from Latin America will be crucial for achieving HIV elimination in this region. Meta-population models could also be relevant for MSM populations in island countries in Asia and the Pacific, which have so far received little attention. Movement and migration [179] will generally become increasingly important as they could aid in importing HIV infections from high-prevalence regions to regions nearing elimination, including in the context of Western countries [4].

Our review also highlights a lack of dynamic modeling studies in regions where there is little HIV prevalence data for MSM. Fig 2B shows no data for much of the Middle East and North Africa (MENA) UNAIDS region. Additionally, we found no dynamic modeling studies for the MENA region. If the previously mentioned barriers (stigma, criminalization, underfunding, etc.) to data collection in this region can be overcome, the potential for modeling studies to be developed in this region will be opened.

In our review, almost all studies considered MSM in Africa as part of a wider population consisting of the heterosexual population and key populations other than MSM [71,101,102,106,112,124–126,133]. In contrast to concentrated epidemics among MSM in Western and Central Europe and North America (e,g. [69,88,132]), HIV epidemics among MSM and the heterosexual population in Africa are substantially mixed due to MSM also having sex with women [180,181]. However, while the numbers of new infections in the overall adult population, sex workers, and their clients in Africa have been falling at the same rate, no such progress has been observed for MSM [3,10]. If MSM are left behind in the HIV response in Africa, they may continue to be a source of infection for women in the general population in the future, potentially undermining the progress made in eliminating HIV within this population. The inclusion of MSM in transmission models for African countries is needed to inform tailored interventions that ensure equal rates of progress towards HIV elimination for different key populations and the general population [166]. Given the proven benefits of PrEP in Western countries and Asia, exploring its impact on HIV elimination among MSM in Africa is desirable. Additionally, while public health literature highlights the benefits of PEP, the population-level impact of this intervention remains largely unexplored in Africa and globally [182], as does the impact of long-acting injectable PrEP compared to oral PrEP. Two studies in our review [47,108] demonstrated that long-acting injectable PrEP programs can achieve HIV elimination at much lower coverage compared to oral PrEP, and another study [131] suggested that long-acting injectable PrEP may efficiently supplement oral PrEP programmes by expanding overall PrEP coverage in high-incidence settings. However, all three studies focused on MSM in Atlanta, Georgia, USA, so these findings may not be generalisable to contexts with different background HIV care continua. Technological innovation in long-acting PrEP has accelerated in recent years, exemplified by the approval of lenacapavir, a twice-yearly injectable PrEP agent that provides an alternative to daily oral PrEP [183]. Mathematical modelling can be used not only to evaluate strategies for the implementation and deployment of these technologies, but also to examine the mechanisms underlying their effectiveness, thereby identifying characteristics that may inform the future development of HIV prevention technologies.

Another modeling direction involves understanding the elimination of disparities in HIV burden among diverse subgroups of MSM. As overall incidence decreases, disparities may be exacerbated [166]. Therefore, eliminating disparities is a parallel goal to achieving the overall target for HIV elimination. In our review, this topic has received much attention in the context of epidemics in the USA [52,54,63,75], where pronounced disparities in HIV burden have been observed due to assortative mixing of racial and ethnic subgroups and differences in their interaction with the healthcare system (e.g., testing rates, ART, and PrEP coverage). However, the issue of disparities is not limited to the USA and is relevant to characteristics other than ethnicity in many countries. For example, substantial age differences in HIV prevalence are observed among MSM in Africa [184] and the Caribbean [9]. On the road to elimination, disparities may increase between native and migrant populations [179], as well as between rural and urban communities. In our review, simpler models with fewer stratifications such as DCMs and SCMs predominated outside Western and Central Europe and North America. More complex ABMs will be required to simulate elimination interventions that are sufficiently nuanced to address the vulnerabilities of diverse subgroups of MSM. The need for improved incorporation of characteristics such as ethnicity in future models was also discussed in other literature [15,161].

Finally, modeling could be useful for understanding how new HIV technologies and biomedical prevention tools might affect transmission dynamics and either strengthen or undermine the feasibility of elimination in different regions. Research efforts are increasingly dedicated to improving the quality of life for people already living with HIV. Biomedical advancements may lead to a potential HIV cure in the future, with a target product profile already formulated [185]. Notably, the target product profile considers the possibility of re-infection and viral rebound after ART-free suppression acceptable. These characteristics imply that introducing an HIV cure may result in new infections among MSM. This implementation of an HIV cure was investigated in a recently published modeling study [26], which showed that the population-level impact of a future HIV cure may vary substantially by cure mechanism and implementation strategy, underscoring the need to identify conditions under which cure introduction could maximise benefit while avoiding unintended increases in HIV incidence among MSM. These modeling considerations are important because HIV cure technologies are expected to have implications beyond viral suppression: both people with HIV and key populations without HIV expect the existence of an HIV cure to have positive effects on quality of life, sexual satisfaction, and stigma [186]. While only one modeling study on HIV cure met our inclusion criteria, the potential for a HIV cure to be developed in the near future may lead this to be a major focus in HIV modeling.

Investigating future scenarios involving such potential interventions will be necessary to guide their implementation in the context of current HIV elimination efforts.

### Limitations

In the absence of prior systematic reviews on the possibility of global elimination among MSM, we focused our study on mathematical transmission models. These models have the advantage of providing a mechanistic understanding of the transmission process and are frequently used to inform policymakers. The synthesis of elimination definitions and interventions that may lead to elimination in a broader class of models available in the literature (e.g., statistical, back-calculation, decision-analytic, health economic) should be a subject of future research. Additionally, we compared the worldwide location of the studied populations with the HIV prevalence among MSM as reported by the UNAIDS key population Atlas [1]. These UNAIDS data are compiled from public sources and reviewed for quality, though the quality may vary. Other recent estimates of HIV prevalence among MSM cover only specific UNAIDS regions (e.g., [9,163,164]) and their use would not alter our overall conclusions about the underrepresentation of MSM populations. However, it is important to note that we did not actively seek studies not published in English and this may have contributed to the finding that some UNAIDS regions are underrepresented in modeling studies. We also did not include studies that modeled MSM populations as part of a generalized HIV epidemic but did not report outcomes specifically for MSM, potentially contributing to our conclusions that modeling studies are underrepresented in certain regions.

Furthermore, the presence of various elimination definitions, intervention scenarios, outcome measures, and model types complicated the comparison of efficient intervention scenarios across settings. Different studies modeled similar interventions using a range of modeling paradigms, making cross-comparison challenging and precluding meaningful quantitative synthesis or meta-analysis. Consequently, our conclusions regarding the potential for HIV elimination are based on a descriptive synthesis of the available modelling evidence and should be interpreted within the context of this heterogeneity.

Although we extracted some details on model structures and methodologies, our review primarily focused on elimination definitions and the potential for elimination through various interventions rather than on the structural composition of the models. Therefore, we did not comment on the suitability of the model structure for projecting elimination prospects beyond the criteria included in the comprehensiveness score and used for critical appraisal of studies. In the future, more research should focus on systematic comparative analyses of different models using consistent elimination definitions, epidemiological outcomes. Although it does not specifically define elimination, the study by Eaton et al. [162] serves as an example of such an analysis. Finally, although we adhered to predefined methodology throughout the review process, the lack of protocol registration may have reduced methodological transparency and introduced potential bias in review decisions. In principle, such changes could affect the studies included, the synthesis of evidence, and the overall conclusions. Therefore, the lack of protocol registration should be considered when interpreting our findings.

## Conclusion

In conclusion, this systematic review compiled evidence on the possibilities of HIV elimination among MSM worldwide by summarizing findings from mathematical modeling studies. There is a need to intensify modeling efforts to assess elimination prospects among MSM outside Western and Central Europe and North America. Additionally, we analyzed the elimination definitions used in current studies and recommended new research directions for modeling to support a coordinated global response to HIV elimination among MSM. Finally, we observe that out of studies that find elimination to be feasible, the overwhelming majority do so when assessing the impact of combinations of interventions, with PrEP being the most significant intervention, both when used independently and in combination.

### Data sharing

The extracted data required for generation of all results, tables and figures are available in S1 Data. The maps in Fig 2A and 2B were generated in R (version 4.5.3) using the rworldmap package, which provides country boundary data derived from the Natural Earth dataset (https://www.naturalearthdata.com/). HIV prevalence data for Fig 2B were acquired from UNAIDS, publically available at: https://kpatlas.unaids.org/dashboard. All other figures were generated in Python (version 3.1.2). All figures and associated code required to generate them is available at https://doi.org/10.5281/zenodo.20400174

## Supporting information

S1 Table Prisma Checklist. This checklist originates from the PRISMA 2020 statement [21]. A template for this checklist can be found at https://www.prisma-statement.org/.
(DOCX)

S1 Data Data table. Table with all data extracted from included articles.
(XLSX)

S2 Table Inclusion and exclusion reasons for searched articles. Inclusion and exclusion decision reasons for all articles identified through the database search.
(XLSX)

S1 Text Additional figures and tables. Additional analyses supplementing the results presented in the main text. Contains S1-S3 Figs and S1-S3 Tables.
(PDF)

S2 Text Model comprehensiveness. Additional tables presenting the framework and scoring of the novel model comprehensiveness tool. Contains S4-S6 Tables.
(PDF)
