# Supplementary material for: Prospects of HIV elimination among men who have sex with men: A systematic review of modeling studies
Source: PLoS Comput Biol. 2026 Aug 31;22(8):e1014596. doi: 10.1371/journal.pcbi.1014596 (PMC13557506; doi:10.1371/journal.pcbi.1014596)
Supplement: Supplementary file 1 [file pcbi.1014596.s004.pdf]

# Supplementary figures and tables

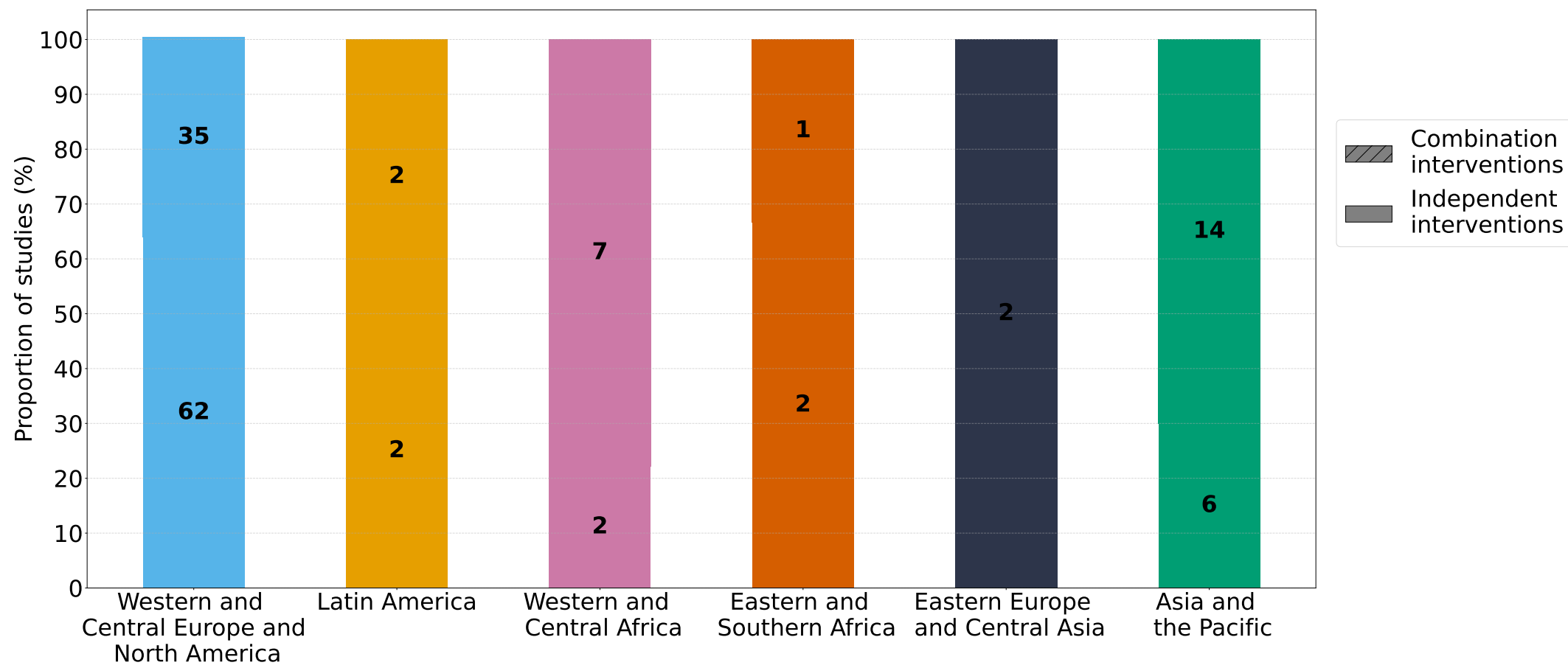


**Fig A. Inclusion of modeled combination and individual interventions in studies.** We refer to individual interventions as interventions that were studied independently. Combination interventions imply that at least two interventions were studied in combination. The labels on each bar represent the number of times interventions were included in combination or independently.

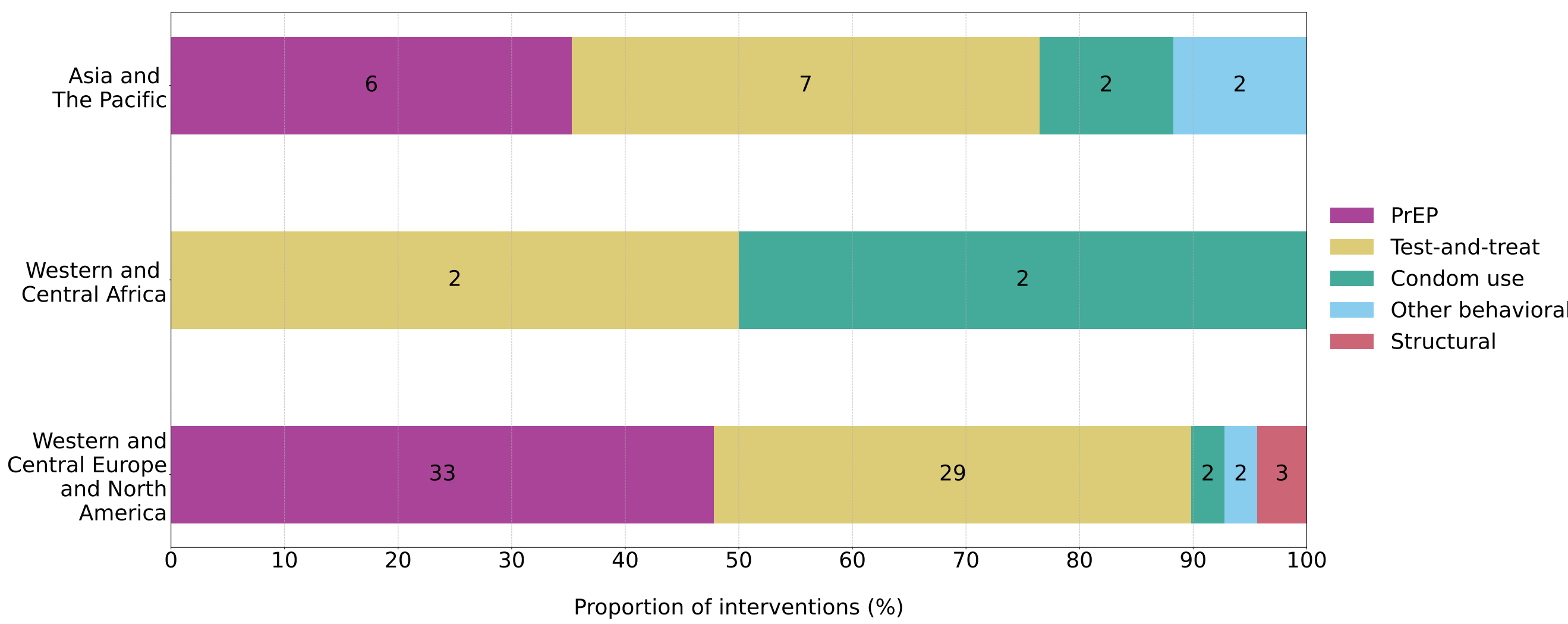


**Fig B. Distribution of modeled interventions by UNAIDS region in studies that defined elimination.** The labels on each bar represent the number of times the intervention was included. A single study might include multiple interventions. Pre-exposure prophylaxis = PrEP.

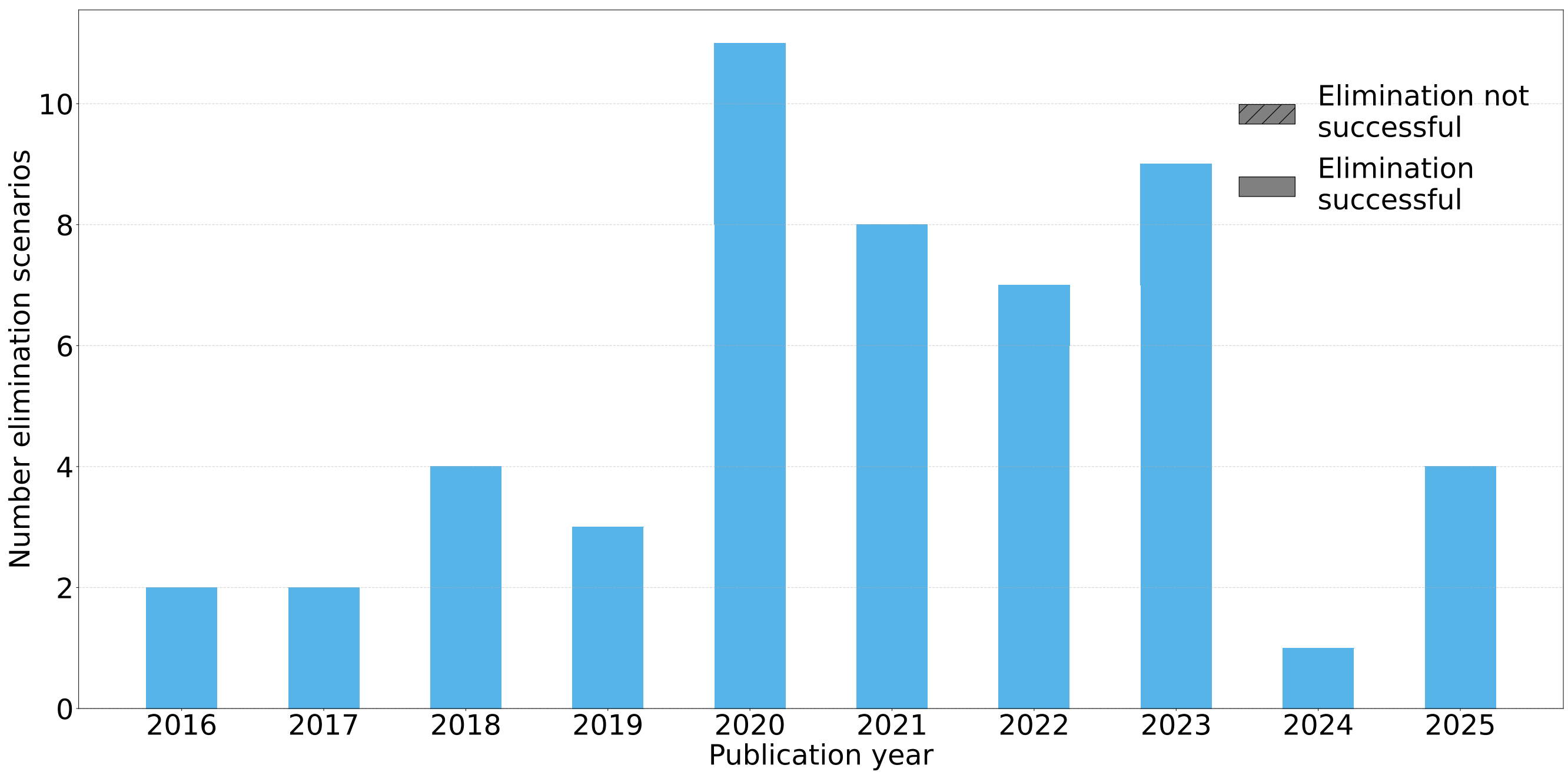


**Fig C. Distribution elimination scenarios across included studies by publication year.** Study search was conducted between July 1 2016 - September 1 2025, so real values for 2016 & 2025 are likely higher. A single study might include multiple definitions. Pre-exposure prophylaxis = PrEP.

**Table A. Achievability of elimination through modeled combination and individual interventions.** ND = not discussed. - = not applicable. UNAIDS = The Joint United Nations Programme on HIV/AIDS.

| **Elimination achievable** | **Interventions** | **Feasibility** | **UNAIDS regions** | | | |
|---|---|---|---|---|---|---|
| | | | Western and Central Europe and North America n (%) | Western and Central Africa n (%) | Asia and The Pacific n (%) | **Total** **n** (%) |
| Yes | Combination | Likely | 3 (13.64) | 0 (0.00) | 4 (66.67) | **7** (24.14) |
| | | Unlikely | 9 (40.91) | 0 (0.00) | 0 (0.00) | **9** (31.03) |
| | | ND | 10 (45.45) | 1 (100.00) | 2 (33.33) | **13** (44.83) |
| | | **Total** | **22** (68.75) | **1** (100.00) | **6** (85.71) | **29** (70.73) |
| | Individual | Likely | 1 (10.00) | 0 (0.00) | 1 (100.00) | **2** (16.67) |
| | | Unlikely | 8 (80.00) | 0 (0.00) | 0 (0.00) | **9*** (75.00) |
| | | ND | 1 (10.00) | 0 (0.00) | 0 (0.00) | **1** (8.33) |
| | | **Total** | **10** (31.25) | **0** (0.00) | **1** (14.29) | **12*** (29.27) |
| | **Total** | | **32** (82.05) | **1** (50.00) | **7** (87.5) | **41*** (82.00) |
| No | Combination | - | 6 (85.71) | 1 (100.00) | 0 (0.00) | **7** (77.78) |
| | Individual | - | 1 (14.29) | 0 (0.00) | 1 (100.00) | **2** (22.22) |
| | **Total** | | **7** (17.95) | **1** (50.00) | **1** (12.25) | **9** (18.00) |
| **Total** | | | **39** | **2** | **8** | **50***,† |

* The study [37] without the reference to a specific location predicted elimination as achievable, but unlikely, using an individual intervention.
† The study [107] was not included in this table because it defined elimination but did not directly assess its achievability.

**Table B. Interventions included in modeled scenarios by achievability of elimination.** UNAIDS = The Joint United Nations Programme on HIV/AIDS.

| **Elimination achievable** | **Intervention** | **UNAIDS region** | | | |
|---|---|---|---|---|---|
| | | Western and Central Europe and North America n (%) | Western and Central Africa n (%) | Asia and The Pacific n (%) | **Total** **n** (%) |
| Yes | PrEP | 26 (44.83) | 0 (0.00) | 7 (38.89) | **32** (40.51) |
| | Test-and-treat | 25 (43.10) | 1 (50.00) | 7 (38.89) | **33*** (41.77) |
| | Condom use | 2 (3.45) | 1 (50.00) | 2 (11.11) | **5** (6.33) |
| | Other behavioral | 2 (3.45) | 0 (0.00) | 2 (11.11) | **4** (5.06) |
| | Structural | 3 (5.17) | 0 (0.00) | 0 (0.00) | **3** (3.80) |
| | **Total** | **58** (84.06) | **2** (50.00) | **18** (94.74) | **79*** (84.95) |
| No | PrEP | 6 (54.55) | 0 (0.00) | 0 (0.00) | **6** (42.86) |
| | Test-and-treat | 5 (45.45) | 1 (50.00) | 1 (100.00) | **7** (50.00) |
| | Condom use | 0 (0.00) | 1 (50.00) | 0 (0.00) | **1** (7.15) |
| | Other behavioral | 0 (0.00) | 0 (0.00) | 0 (0.00) | **0** (0.00) |
| | Structural | 0 (0.00) | 0 (0.00) | 0 (0.00) | **0** (0.00) |
| | **Total** | **11** (15.94) | **2** (50.00) | **1** (5.26) | **14** (15.05) |
| **Total** | | **69** | **4** | **19** | **93*** |

* The study [37] did not reference a specific location but did predict elimination as achievable using test-and-treat.
The study by Milwid et al. [107] defined elimination but did not assess any elimination scenarios and, therefore, was not counted in this table.

**Table C. Achievability of elimination by type of modeled intervention.** UNAIDS = The Joint United Nations Programme on HIV/AIDS.

| **Intervention** | **Elimination achievable** | **UNAIDS region** | | | |
|---|---|---|---|---|---|
| | | Western and Central Europe and North America n (%) | Western and Central Africa n (%) | Asia and The Pacific n (%) | **Total** **n** (%) |
| PrEP | Yes | 26 (81.25) | 0 (0.00) | 7 (100.00) | **33** (84.62) |
| | No | 6 (18.75) | 0 (0.00) | 0 (0.00) | **6** (15.38) |
| | **Total** | **32** (46.38) | **0** (0.00) | **7** (36.84) | **39** (41.94) |
| Test-and-treat | Yes | 25 (83.33) | 1 (50.00) | 7 (87.50) | **34*** (77.27) |
| | No | 5 (16.67) | 1 (50.00) | 1 (12.50) | **7** (22.73) |
| | **Total** | **30** (40.58) | **2** (50.00) | **8** (42.11) | **41*** (44.09) |
| Condom use | Yes | 2 (100.00) | 1 (50.00) | 2 (100.00) | **5** (83.33) |
| | No | 0 (0.00) | 1 (50.00) | 0 (0.00) | **1** (16.67) |
| | **Total** | **2** (2.90) | **2** (50.00) | **2** (10.53) | **6** (6.45) |
| Other behavioral | Yes | 2 (100.00) | 0 (0.00) | 2 (100.00) | **4** (100.00) |
| | No | 0 (0.00) | 0 (0.00) | 0 (0.00) | **0** (0.00) |
| | **Total** | **2** (2.90) | **0** (0.00) | **2** (10.53) | **4** (4.30) |
| Structural | Yes | 3 (100.00) | 0 (0.00) | 0 (0.00) | **3** (100.00) |
| | No | 0 (0.00) | 0 (0.00) | 0 (0.00) | **0** (0.00) |
| | **Total** | **3** (4.35) | **0** (0.00) | **0** (0.00) | **3** (3.23) |
| **Total** | | **69** | **4** | **19** | **93*** |

* The study [37] did not reference a specific location but did predict elimination as achievable using test-and-treat.
The study by Milwid et al. [107] defined elimination but did not assess an elimination scenario and, therefore, was not counted in this table.
