# Supplementary material for: Prospects of HIV elimination among men who have sex with men: A systematic review of modeling studies
Source: PLoS Comput Biol. 2026 Aug 31;22(8):e1014596. doi: 10.1371/journal.pcbi.1014596 (PMC13557506; doi:10.1371/journal.pcbi.1014596)
Supplement: Supplementary file 2 [file pcbi.1014596.s005.pdf]

Model comprehensiveness

Table A. Comprehensiveness criteria.

Criterion	Considerations	Score considerations
Adherence to interventions	Does the model include parameters to account for adherence to the primary interventions?	0: Not included for any primary interventions. 0.5: Included for some but not all primary interventions. 1: Included for all primary interventions.
Sexual risk compensation	Does the model include parameters to account for changes in sexual behavior as a result of interventions?	0: Not included. 1: Included.
Openness of the MSM population	Does the model account for the introduction of new individuals to the population who have HIV? Does the model allow for mixing between MSM and other subpopulations, such as the heterosexual population?	0: No introduction of individuals with HIV or mixing between MSM and other subpopulations. 1: At least one of these considerations included.
Uncertainty in model outcomes	Does the model report results with appropriate uncertainty, such as simulation intervals or uncertainty from parameter sets? Have any sensitivity analyses been performed with respect to parameters that were estimated or assumed?	0: No sensitivity analyses and no uncertainty reported in the results. 0.5: One of these considerations included. 1: Both considerations included.
Model validation	Is there an attempt made to validate the model outputs against existing data?	0: No attempt made. 0.5: Informal attempt made, i.e, details are unclear or validation performed against empirically derived targets. 1: Clear validation carried out.

* Adherence to interventions: Adherence can vary by population geography and demographics, leading to variations in real-world effectiveness in different epidemiological contexts [187].
Sexual risk compensation: A well studied phenomenon that increasing intervention coverage can lead to a counterintuitive increase in incidence or diminishing returns [188].
Openness of the MSM population: International migrants have a higher HIV prevalence than native-born populations [179], when incidence is low in a country then imported infections become significant in the context of elimination.
Uncertainty in model outcomes: A study which does not take into account any uncertainty might be overconfident in predicting elimination [189].
Model validation: Validating models against existing data gives confidence that the model outputs are grounded in reality [190].

Table B. Model comprehensiveness scoring.

Authors (year)	Adherence to interventions	Sexual risk compensation	Openness of the MSM population	Uncertainty in model outcomes	Model validation	Overall score
Cambiano et al (2023) [32]	1	0	1	1	0	**3**
Chen et al (2022) [35]	1	0	1	1	1	**4**
David et al (2020) [37]	0	0	0	0.5	0	**0.5**
Fojo et al (2021) [45]	1	0	1	1	0	**3**
Fraysse et al (2025)* [47]	1	1	0	0	1	**3**
Gilmour et al (2020)* [49]	0.5	0	0	1	0	**1.5**
Le Guillou et al (2021) [57]	1	1	0	0.5	0	**2.5**
Gutowska et al (2022) [59]	1	0	0	1	0	**2**
Hamilton et al (2023) [18]	1	0	1	0.5	0	**2.5**
Hansson et al (2020) [64]	1	0	0	1	0	**2**
Hansson et al (2019)* [65]	1	0	0	1	0	**2**
Jenness et al (2020) [74]	1	1	0	1	0.5	**3.5**
Jijon et al (2021) [78]	1	1	0	1	0	**3**
Khanna et al (2021) [85]	1	0	1	1	0	**3**
Khanna et al (2019) [86]	1	0	1	1	0	**3**
Lee et al (2023) [90]	1	0	1	1	0.5	**3.5**
Lee et al (2022) [91]	1	0	1	1	0.5	**3.5**
Li et al (2018)* [92]	1	1	0	1	0	**3**
Lima et al (2021) [93]	1	1	0	1	1	**4**
Lou et al (2017) [97]	1	1	0	1	0	**3**
Lu et al (2020) [99]	1	0	0	1	1	**3**
Maheou-Giroux et al (2017) [101]	1	0	1	1	0	**3**
Martin et al (2020) [105]	1	0	0	0.5	0.5	**2**
Massey et al (2023) [104]	0.5	0	1	0	0	**1.5**
Milwid et al (2022) [107]	1	0	0	0.5	1	**2.5**
Mitchell et al (2023) [108]	1	0	0	1	1	**3**
Mitchell et al (2019) [110]	1	0	0	1	1	**3**
Mitchell et al (2016)* [111]	1	1	1	0.5	1	**4.5**
Palk et al (2018)* [4]	1	0	1	1	0	**3**
Rozhnova et al (2018)* [16]	1	0	0	1	0	**2**
Rozhnova et al (2016) [123]	1	0	0	1	0	**2**
Silhol et al (2021) [125]	1	0	1	1	0.5	**3.5**
Singleton et al (2020) [128]	1	0	0	1	0	**2**
Tollett et al (2024) [135]	0.5	0	0	1	1	**2.5**
Vermeer et al (2022) [138]	1	0	0	1	1	**3**
Wang et al (2018) [145]	1	0	0	1	0	**2**
Wang et al (2025) [142]	1	0	0	1	0	**2**
Wang et al (2022)* [143]	0.5	0	0	1	0	**1.5**
Wheatly et al (2022) [147]	1	1	0	1	1	**4**
Wu et al (2025)* [148]	1	1	0	0.5	0	**2.5**
Zang et al (2023) [149]	0.5	0	1	0	1	**2.5**

* These studies predicted elimination in the modeled intervention scenarios, which were also discussed as being plausible in a real-world context.

Table C. Comprehensiveness score distribution by model type.

Score	Model type			
	ABM	DCM	SCM	**Total**
	n (%)	n (%)	n (%)	n (%)
0.0	0 (0.00)	0 (0.00)	0 (0.00)	**0** (0.00)
0.5	0 (0.00)	1 (4.17)	0 (0.00)	**1** (2.44)
1.0	0 (0.00)	0 (0.00)	0 (0.00)	**0** (0.00)
1.5	0 (0.00)	2 (8.33)	1 (25.00)	**0** (0.00)
2.0	1 (7.69)	7 (29.17)	1 (25.00)	**9** (21.95)
2.5	3 (23.08)	2 (8.33)	1 (25.00)	**6** (14.63)
3.0	5 (38.46)	8 (33.33)	1 (25.00)	**14** (34.15)
3.5	3 (23.08)	1 (4.17)	0 (0.00)	**4** (9.76)
4.0	1 (7.69)	2 (8.33)	0 (0.00)	**3** (7.32)
4.5	0 (0.00)	1 (4.17)	0 (0.00)	**1** (2.44)
5.0	0 (0.00)	0 (0.00)	0 (0.00)	**0** (0.00)
**Total**	**13**	**24**	**4**	**41**
**Average score**	**3.0**	**2.81**	**2.25**	**2.59**
